# Deficiency of *N*-glycanase 1 perturbs neurogenesis and cerebral development modeled by human organoids

**DOI:** 10.1038/s41419-022-04693-0

**Published:** 2022-03-24

**Authors:** Victor J. T. Lin, Jiangnan Hu, Ashwini Zolekar, Max R. Salick, Parul Mittal, Jordan T. Bird, Peter Hoffmann, Ajamete Kaykas, Stephanie D. Byrum, Yu-Chieh Wang

**Affiliations:** 1 Department of Pharmaceutical Sciences, UNT System College of Pharmacy, University of North Texas Health Science Center, Fort Worth, TX USA; 2 Department of Dermatology, Medical College of Wisconsin, Milwaukee, WI USA; 3 Department of Neuroscience, Novartis Institutes for Biomedical Research, Cambridge, MA USA; 4 Future Industries Institute, University of South Australia, Adelaide, SA Australia; 5 Department of Biochemistry and Molecular Biology, University of Arkansas for Medical Sciences, Little Rock, AR USA

**Keywords:** Developmental neurogenesis, Neural stem cells, Neuronal development, Stress and resilience, Developmental disorders

## Abstract

Mutations in *N*-glycanase 1 (NGLY1), which deglycosylates misfolded glycoproteins for degradation, can cause NGLY1 deficiency in patients and their abnormal fetal development in multiple organs, including microcephaly and other neurological disorders. Using cerebral organoids (COs) developed from human embryonic stem cells (hESCs) and induced pluripotent stem cells (hiPSCs), we investigate how NGLY1 dysfunction disturbs early brain development. While NGLY1 loss had limited impact on the undifferentiated cells, COs developed from NGLY1-deficient hESCs showed defective formation of SATB2-positive upper-layer neurons, and attenuation of STAT3 and HES1 signaling critical for sustaining radial glia. Bulk and single-cell transcriptomic analysis revealed premature neuronal differentiation accompanied by downregulation of secreted and transcription factors, including TTR, IGFBP2, and ID4 in NGLY1-deficient COs. NGLY1 malfunction also dysregulated ID4 and enhanced neuronal differentiation in CO transplants developed in vivo. NGLY1-deficient CO cells were more vulnerable to multiple stressors; treating the deficient cells with recombinant TTR reduced their susceptibility to stress from proteasome inactivation, likely through LRP2-mediated activation of MAPK signaling. Expressing NGLY1 led to IGFBP2 and ID4 upregulation in CO cells developed from NGLY1-deficiency patient’s hiPSCs. In addition, treatment with recombinant IGFBP2 enhanced ID4 expression, STAT3 signaling, and proliferation of NGLY1-deficient CO cells. Overall, our discoveries suggest that dysregulation of stress responses and neural precursor differentiation underlies the brain abnormalities observed in NGLY1-deficient individuals.

## Introduction

*N*-glycanase 1 (NGLY1) is a glycoenzyme that facilitates proteasome-mediated protein degradation by removing *N*-glycans from denatured glycoproteins [1–5]. Mutations in the *NGLY1* gene can lead to dysfunctional NGLY1 and cause a congenital deglycosylation disorder, NGLY1 deficiency [5, 6]. Patients with NGLY1 deficiency often present with global developmental delay accompanied by neurological symptoms, including microcephaly [3, 5, 7, 8]. Similar to the patients, *Ngly1*-knockout rats exhibit multiple neuropathological phenotypes [9]. Flies and worms with defective *NGLY1* orthologs also show developmental retardation and unusual axon branching [10–13]. Although the clinical findings and animal model phenotypes indicate a critical role NGLY1 plays in neurodevelopment and neurophysiology, how NGLY1 dysfunction causes neurodevelopmental abnormalities in a human-relevant system remains largely unknown.

Loss-of-function studies in different cell types have begun to uncover a few pathways by which NGLY1 modulates cellular activities. NGLY1 inactivation disrupts NFE2L1 function and potentiates proteasome inhibitor toxicity in myeloma cells [14]. NGLY1 deficiency also causes chronic impairment of mitochondrial and immune homeostasis in mouse fibroblasts and human lymphoblast-like cells [15]. Additionally, NGLY1 inactivation triggers stress response-mediated cell death and upregulation of anticancer interferons in human melanoma cells but not normal dermal fibroblasts [16]. These findings indicate that NGLY1 dysfunction could induce differential responses in cells, depending on their lineage, developmental stages, or perturbations to homeostasis.

While several animal models for NGLY1 deficiency exist, noticeable variability exhibits in observed neurodevelopment phenotypes across taxa [5, 6, 9, 10, 12, 17], suggesting that NGLY1-deficiency effects on human neurogenesis may not be fully recapitulated in animal-only models. A high similarity between human cerebral organoids (COs) and the human fetal neocortex at the transcriptomic level [18] qualifies COs as a relevant model for studying the molecular and cellular features of human cerebral development. Many seminal discoveries on the development, pathogenesis, and pharmacological responses of the central nervous system have been made using COs [19–30]. To understand defective neurodevelopment in NGLY1-deficient individuals, we seek to investigate how NGLY1 malfunction influences human cells undergoing neurogenesis modeled by COs.

Our work uncovers cellular and molecular alterations due to NGLY1 loss in COs developed from human pluripotent stem cells (hPSCs). While viability and cellular pluripotency appear unaffected in NGLY1-deficient hPSCs, NGLY1 dysfunction substantially reduces upper-layer neurons in COs. Accompanied by enhanced stress susceptibility and differentiation propensity, secreted factor and neural stem cell (NSC) signaling in NGLY1-deficient CO cells is hindered in vitro and in vivo. Treatment with recombinant TTR and IGFBP2, two secreted factors highly reduced in NGLY1-deficient COs, promotes the tolerability of the deficient cells to bortezomib-induced proteotoxic stress and helps preserve their ID4 and STAT3 activities that are critical for maintaining NSCs during cerebral development.

## Materials and methods

### Cell culture

WA09-C3, -C4, and -C6 human embryonic stem cells (hESCs) were generated through CRISPR-Cas9-mediated editing of the *NGLY1* gene in WA09 hESCs followed by single-cell cloning [16]. Human-induced pluripotent stem cells (hiPSCs) were established from NGLY1-deficiency patients’ fibroblasts (GM25990 and GM26607; Coriell Institute for Medical Research) using CytoTune Sendai Reprogramming Kit (Thermo Fisher Scientific). Except using TeSR-E8 medium (Stemcell Technologies) and 2 mM EDTA passaging solution (Thermo Fisher Scientific), we generally followed the reported method [31] to culture undifferentiated hPSCs in a feeder cell-free condition. All the hPSCs were routinely subcultured when cell density reached 80% to 90%. The passage numbers of the hESCs and hiPSCs used in our studies spanned across 65–95 and 35–65, respectively. Additional information relevant to the cells was summarized in Table S1. The experiments using hPSCs were performed in compliance with the guidelines and approval of the institutional biosafety committee and embryonic stem cell research oversight committee. All cells were periodically tested using the MycoAlert mycoplasma detection kit (Lonza) and free of mycoplasma.

### Whole-exome sequencing (WES)

WES followed by single nucleotide polymorphism (SNP) analysis was completed through a contracted service at Novogene. The experimental procedures were summarized in Supplementary Information.

### Expression of exogenous NGLY1

A pLenti expression vector that carries a FLAG-tagged NGLY1 open reading frame (OriGene Technologies) was used for exogenous NGLY1 expression driven by a CMV promoter. An empty vector was used as the transduction control.

### Embryoid body (EB) formation

The experimental procedure for non-directed cell differentiation in EBs was summarized in Supplementary Information.

### CO formation

The protocol for CO formation was optimized in our reported study [32] and summarized in Supplementary Information.

### Immunofluorescence staining

The general procedures for immunofluorescence staining were provided in Supplementary Information. Primary antibody information was summarized in Table S2.

### Optical clearing, staining, and imaging in COs

The protocol for CUBIC (Clear, Unobstructed Brain/Body Imaging Cocktails and Computational) analysis was adopted from a previous study [33], optimized for COs, and summarized in Supplementary Information. Primary antibody information was summarized in Table S2.

### Immunoblotting

The procedure was previously described [32]. Primary antibody information was summarized in Table S2.

### Cell viability tests

The procedures were summarized in Supplementary Information.

### Gene expression analysis by microarrays and qRT-PCR

The procedures were provided in Supplementary Information. The PluriTest [34] was used to test cellular pluripotency based on the transcriptomic features of cell samples.

### Single-cell RNA sequencing (scRNA-seq)

Cultrex organoid harvesting solution (R&D Systems) was used to release COs from residual Matrigel envelopes. The released COs were dissociated using a papain-based neural tissue dissociation kit on a gentleMACS dissociator (Miltenyi Biotec). The information of reagents and procedures for library preparation and sequencing based on the Chromium single-cell gene expression pipeline (10x Genomics) was summarized in Supplementary Information. The Seurat package [35] in R was used to analyze the filtered, aggregated, and depth-normalized transcript counts generated by the *cellranger count* and *cellranger aggr* scripts. Additional information for our sequencing data analysis was provided in Supplementary Information.

### Proteomics analysis

The procedures for peptide mass spectrometry and data analysis were summarized in Supplementary Information.

### In vivo studies

The surgical procedure for organoid transplantation was summarized in Supplementary Information. All animal studies were performed under approval from the Institutional Animal Care and Use Committee.

### Statistical analysis

Quantitative data for each test were established from biological replicates (*n* ≥ 3) and presented as mean ± standard deviation. The sample size was chosen based on our experience in prior studies with similar experimental methods, where we frequently found samples of 3–5 replicates gave enough statistical power to detect a significant difference. The significance of differences was primarily determined by the two-tailed Student’s *t*-test for means between two groups or by the logistic regression for testing categorical variables.

## Results

### Cellular pluripotency and vitality remain intact in NGLY1-deficient hPSCs

**Fig. 1:**
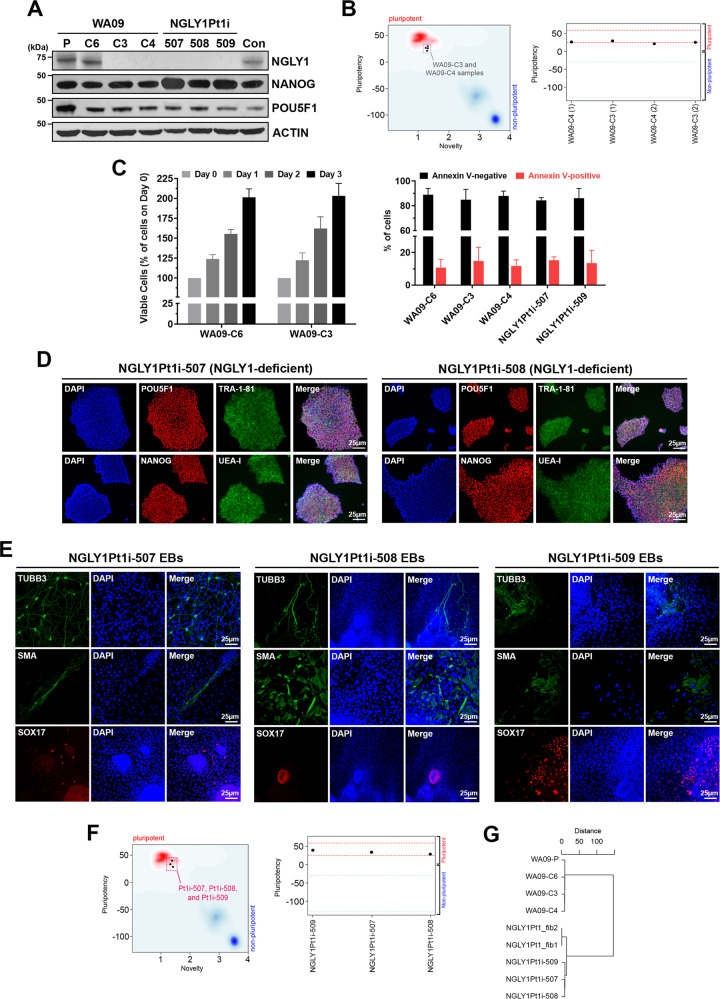
WA09 hESCs with CRISPR-Cas9-introduced NGLY1 mutations and hiPSCs reprogrammed from NGLY1-deficiency patient’s fibroblasts are viable and pluripotent. **A** NGLY1, NANOG, and POU5F1 protein expression detected by western blotting in hESCs and hiPSCs. *WA09-P:* parental WA09 hESCs. *WA09-C6:* a WA09 hESC clone that went through the gene-editing process but acquired no mutation in the *NGLY1* gene. *WA09-C3 and WA09-C4*: NGLY1-deficient WA09 hESC clones derived from gene editing. *NGLY1Pt1i*: patient-derived hiPSCs. *Con:* normal individual−derived control hiPSCs. **B** WA09-C3 and -C4 hESCs were tested pluripotent in the Pluritest. **C** Cell proliferation and apoptosis in undifferentiated samples of the indicated hPSCs determined by MTT (*left panel*) and annexin V staining−mediated flow cytometry (*right panel*) analysis. All data were presented as mean ± standard deviation (*n* = 4). **D** The staining of pluripotency markers, including TRA-1-81, UEA-I, POU5F1, and NANOG, in NGLY1Pt1i-507 and NGLY1Pt1i-508 hiPSCs. **E** EBs containing cells relevant to three germ-layer lineages were developed from the patient-derived hiPSCs. *TUBB3:* an ectoderm marker. *SMA:* a mesoderm marker. *SOX17:* an endoderm marker. **F** The patient-derived hiPSCs were tested pluripotent in the Pluritest. **G** The clustering result among the indicated samples was based on their SNPs detected by WES. *NGLY1Pt1_fib1 and NGLY1Pt1_fib2:* NGLY1-deficiency patient’s skin fibroblast samples.

NGLY1-deficient WA09-C3 and WA09-C4 cells (Fig. 1A) were generated by gene editing in WA09 hESCs with wild-type NGLY1 [16]. DNA sequencing confirmed their deletion mutations of the *NGLY1* gene (Supplementary Fig. S1). While WA09-C6 hESCs also went through the editing process, they escaped from editing and retained NGLY1 expression comparable to that of parental cells (Fig. 1A). WA09-C3 and WA09-C4 hESCs were tested pluripotent by the PluriTest (Fig. 1B). They also formed EBs with differentiated cells relevant to three germ layers [16]. In addition, NGLY1 loss did not affect the overall vitality of undifferentiated WA09 hESCs (Fig. 1C).

NGLY1-deficiency patient’s fibroblasts were reprogrammed into hiPSCs [16]. The patient−derived hiPSCs, including NGLY1Pt1i-507, NGLY1Pt1i-508, NGLY1Pt1i-509, and NGLY1Pt2i-502 cells, expressed multiple markers for cellular pluripotency (Fig. 1D; Supplementary Fig. S2) [16]. They developed EBs with differentiated cells relevant to three germ layers (Fig. 1E; Supplementary Fig. S2) and were tested pluripotent also by the PluriTest (Fig. 1F). Through WES, we found the SNP profiles of our NGLY1-functional and -deficient hESCs are highly similar, clustered altogether, and distant from those of NGLY1-deficiency patient’s fibroblasts and hiPSCs (Fig. 1G). This result further supports that our NGLY1-functional and -deficient hESCs are virtually identical, except their NGLY1 conditions.

### Upper-layer neuron formation and signaling critical for radial glia are disrupted in NGLY1-deficient COs

**Fig. 2:**
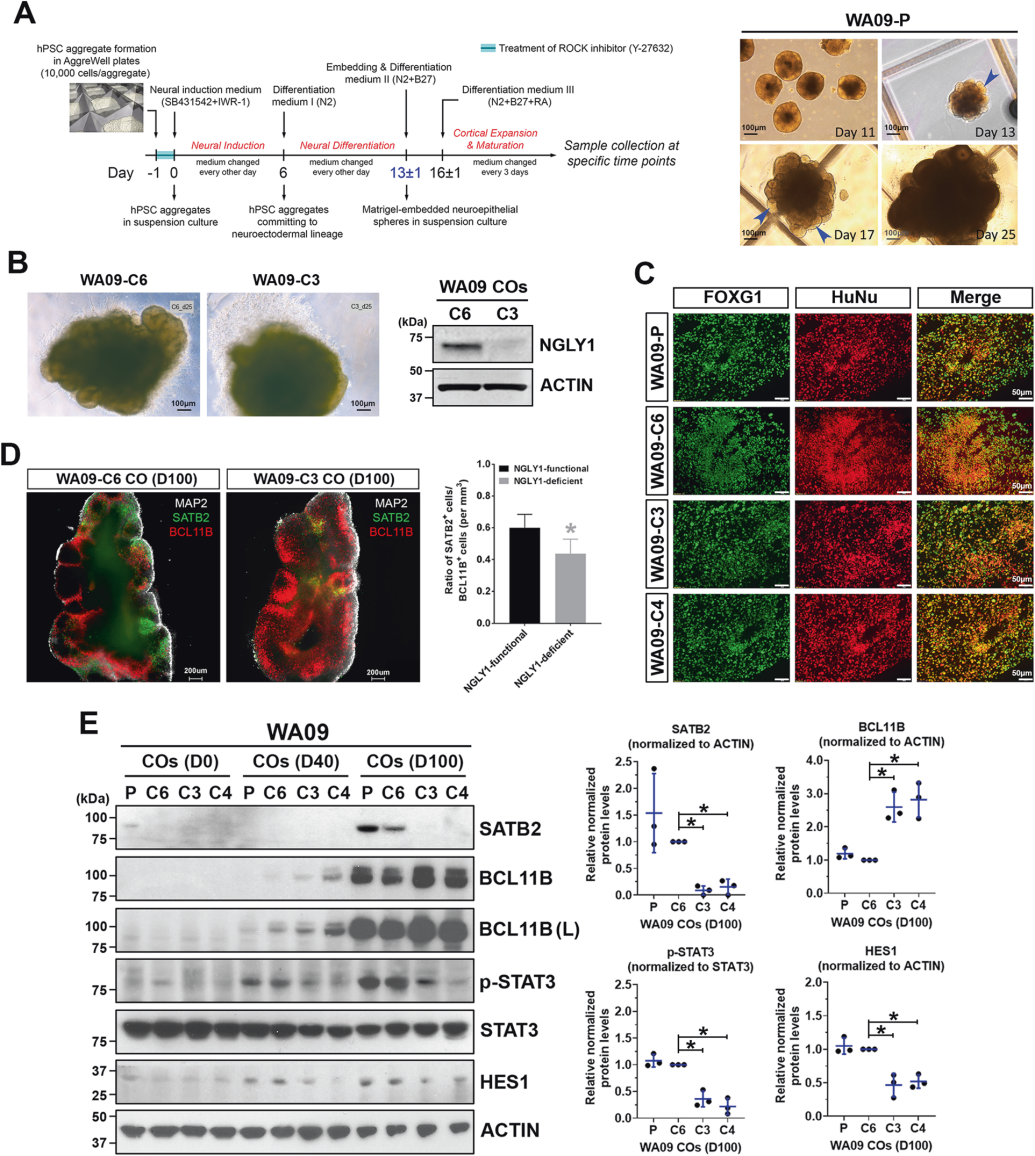
NGLY1-deficient COs differ from NGLY1-functional COs in their capacity to form upper-layer neurons and show alterations in critical signaling needed for radial glia. **A** The schematic illustration of the protocol for CO development (*left panel*) used in our studies and the morphological representations of WA09-P COs at the indicated time points along development (*right panel*). *Blue arrowheads:* neuroepithelial buds, *WA09-P:* parental WA09 hESCs. **B** The representative morphology of NGLY1-functional and -deficient WA09 hESCs COs with 25 days of development. The expression of NGLY1 in the WA09-C6 (NGLY1-functional) and WA09-C3 (NGLY1-deficient) COs was confirmed by western blotting. **C** Both NGLY1-functional and -deficient COs positively stained for forebrain marker FOXG1 and human nuclear antigen. *WA09-P and WA09-C6:* NGLY1-functional. *WA09-C3 and WA09-C4:* NGLY1-deficient. **D** The representative images of optical sections taken for CO samples with SATB2, BCL11B and MAP2 staining; upper-layer (SATB2+) neurons and deeper-layer (BCL11B+) neurons (*left panel*). The ratios of SATB2+ and BCL11B+ cell counts in COs (*n* = 4 for each NGLY1 condition, *p < 0.05, *t*-test) for each NGLY1 condition (*right panel*). *WA09-C6 CO:* NGLY1-functional CO. *WA09-C3 CO:* NGLY1-deficient CO. *D100:* 100 days of CO development. **E** The expression of SATB2, BCL11B, phosphorylated STAT3, and HES1 detected by western blotting with densitometry analysis in NGLY1 functional and -deficient COs with 0, 40, and 100 days (D0, D40, and D100) of development. *P and C6:* NGLY1-functional COs. *C3 and C4:* NGLY1-deficient COs. *(L):* long exposure. *p < 0.05, *t*-test.

Using a protocol depicted in Fig. 2A, we generated COs from NGLY1-functional and -deficient hESCs (Fig. 2B). Having FOXG1-positive cells similarly present in both CO types (Fig. 2C) indicated that NGLY1 deficiency does not affect telencephalic commitment in neuroectoderm cells. However, compared with NGLY1-functional COs, the deficient ones appeared to have more BCL11B-positive deeper-layer neurons after 40-100 days of development (Fig. 2D, E). In contrast, SATB2-positive upper-layer neurons were significantly fewer in the deficient COs (Fig. 2D; Supplementary information, Vid. S1 and S2). These findings suggest that NGLY1 dysfunction may perturb migration and differentiation of progenitor cells into upper-layer neurons.

Because radial glial cells (RGCs) not only comprise cytoarchitectural scaffolding for cell migration and layering but also serve as progenitors that give rise to many different types of neurons in the developing brain [36–38], we examined the activity of STAT3 and HES1 that are critical for RGC maintenance [39, 40]. NGLY1 loss diminished STAT3 phosphorylation and HES1 expression in COs (Fig. 2E), suggesting that RGCs could be disturbed and prone to differentiation in response to NGLY1 malfunction.

### NGLY1 dysfunction enhances a propensity for neuronal differentiation in COs

**Fig. 3:**
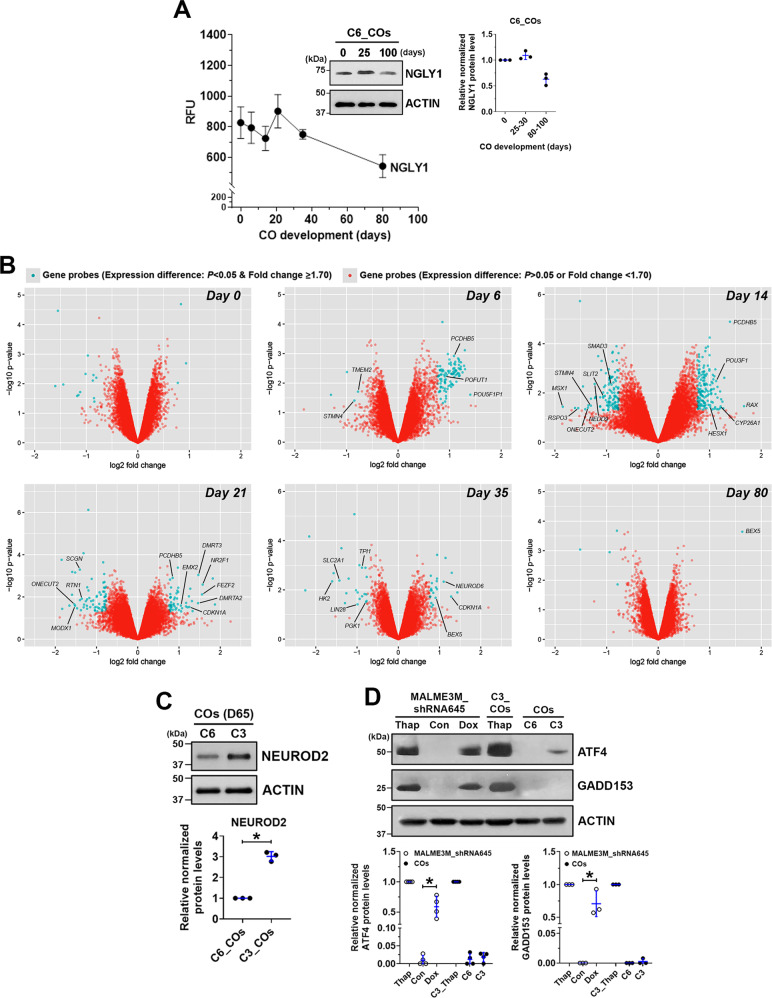
Microarray-based gene expression profiling reveals the propensity for premature differentiation in NGLY1-deficient COs compared with NGLY1-functional COs. **A** The expression pattern of the *NGLY1* gene transcripts in WA09-P and WA09-C6 COs (*n* = 4) at six time points during 80-day development. *Inset:* The relative expression of NGLY1 protein detected by western blotting with densitometry analysis in WA09-C6 COs at the indicated time points. **B** Genes that were differentially expressed (*p* < 0.05, fold change ≥1.7) in NGLY1-functional and -deficient COs at the indicated time points for sample collection were highlighted in blue in the volcano plots of fold change vs. statistical significance. **C** The differential expression of NEUROD2 detected by western blotting with densitometry analysis in NGLY1-functional and -deficient COs with 65 days (D65) of development. *p < 0.05, *t*-test. **D** The relative expression of ATF4 and GADD153 detected by western blotting with densitometry analysis in NGLY1-functional and -deficient COs and MALME3M_shRNA645 melanoma cells with doxycycline-induced knockdown of NGLY1. *C6:* NGLY1-functional COs. *C3:* NGLY1-deficient COs. *Thap:* 24-h treatment of 0.5 µM thapsigargin. *p < 0.05, *t*-test.

Gene expression in COs at multiple time points was profiled using microarrays. NGLY1 expression was found throughout a normal course of CO development, with relatively stable expression in the initial 30 days and followed by a moderate downregulation but preserved expression until 80 days (Fig. 3A). NGLY1-functional and -deficient hESCs downregulated POU5F1 and NANOG as well as upregulated PAX6 and FOXG1 in highly similar kinetics during CO formation (Supplementary Fig. S3), suggesting that NGLY1 dysfunction would not hinder differentiation of pluripotent cells and their telencephalic development. Differentially expressed genes due to NGLY1 dysfunction were found primarily in day-14 and -21 COs (Fig. 3B). Very few genes with significantly altered expression were identified in either the undifferentiated hESCs or day-80 COs with defective NGLY1 (Fig. 3B). These findings suggest that the dynamic transition of human cells as they exit the pluripotent state and commit to the neural lineage would be highly susceptible to NGLY1 deficiency. In addition, cells in a relatively mature or stable condition, such as differentiated neurons or undifferentiated hPSCs, may be less vulnerable to NGLY1 malfunction.

Among the differentially expressed genes between NGLY1-functional and -deficient COs throughout 80 days of development, genes associated with neuronal differentiation and patterning, including *POU3F1*, *HESX1*, *DMRT3, DMRTA2*, *FEZF2*, and *NEUROD6*, were significantly upregulated more in NGLY1-deficient COs, while several NSC-relevant genes, such as *SLIT2*, *STMN4*, *RTN1*, and *LIN28*, were further downregulated (Fig. 3B). Compared with NGLY1-functional COs, the deficient COs at day 35 also had significant downregulation of genes, such as *HK2*, *SLC2A1*, *PGK1*, and *TPI1* (Fig. 3B), that are known to be downregulated during metabolic transition in neurons as they differentiate from progenitors [41]. After 2-month development, the expression of a post-mitotic neuronal marker, NEUROD2, was higher in the NGLY1-deficient COs (Fig. 3C). When we examined six marker genes that are frequently used to identify neuronal differentiation, NGLY1-deficient COs began to express these markers earlier (Supplementary Fig. S4). Taken together, NGLY1-deficient human cells undergoing neurogenesis are likely to prematurely differentiate and have enhanced neuron formation.

### NGLY1 dysfunction alters stress susceptibility of CO and EB cells

**Fig. 4:**
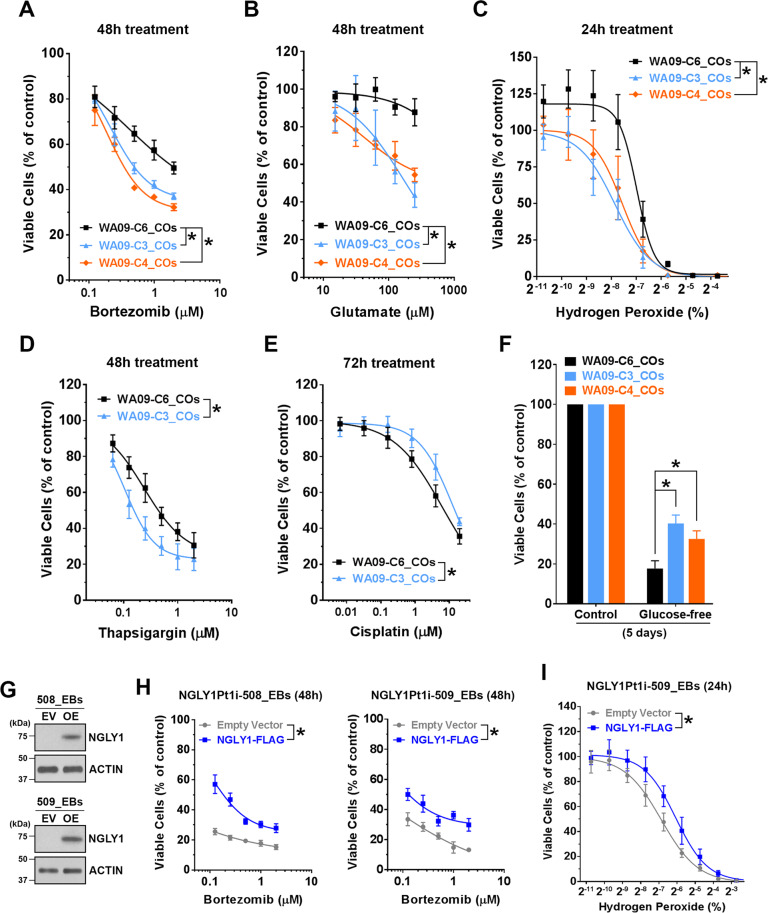
NGLY1 expression alteration within CO and EB cells affects their tolerability to different cell stressors. COs collected at day 60 of development were dissociated to obtain CO cells. Cell viability was determined by MTT assays. **A** The viability of CO cells in response to bortezomib treatment. All data were presented as mean ± standard deviation (*n* = 3, *p < 0.05, logistic regression). *WA09*-*P and WA09-C6:* NGLY1-functional COs. WA09-*C3 and WA09-C4:* NGLY1-deficient COs. **B** The viability of CO cells in response to glutamate treatment. All data were presented as mean ± standard deviation (*n* = 3, *p < 0.05, logistic regression). **C** The viability of CO cells in response to hydrogen peroxide treatment. All data were presented as mean ± standard deviation (*n* = 3, *p < 0.05, logistic regression). **D** The viability of CO cells in response to thapsigargin treatment. All data were presented as mean ± standard deviation (*n* = 3, *p < 0.05, logistic regression). **E** The viability of CO cells in response to cisplatin treatment. All data were presented as mean ± standard deviation (*n* = 3, *p < 0.05, logistic regression). **F** The viability of CO cells in response to glucose deprivation. All data were presented as mean ± standard deviation (*n* = 3, *p < 0.05, *t*-test). EBs cultured for 14 days were dissociated to obtain EB cells for the transduction of the indicated expression vectors. **G** The expression of exogenous NGLY1 in the EB cells developed from NGLY1-deficiency patient−derived (NGLY1Pt1i-508 and NGLY1Pt1i-509) hiPSCs was detected by western blotting. *EV:* Empty/control vector. *OE:* NGLY1-overexpression vector. **H** The reduced susceptibility to the 48-h treatment of bortezomib in NGLY1Pt1i-508 and NGLY1Pt1i-509 EB cells with ectopic NGLY1 expression. All data were presented as mean ± standard deviation (*n* = 3, *p < 0.05, logistic regression). **I** The reduced susceptibility to the 24-h treatment of hydrogen peroxide in NGLY1Pt1i-508 and NGLY1Pt1i-509 EB cells with ectopic NGLY1 expression. All data were presented as mean ± standard deviation (*n* = 3, *p < 0.05, logistic regression).

Having observed ATF4 and GADD153 activation in NGLY1-suppressed melanoma cells [16], we tested whether the relevant signaling similarly alters in NGLY1-deficient COs. While NGLY1 malfunction appeared to upregulate ATF4 in WA09 COs, this upregulation was moderate, compared with that in NGLY1-knockdown melanoma cells (Fig. 3D). Unlike NGLY1-knockdown melanoma cells, NGLY1-deficient COs showed undetectable GADD153 protein (Fig. 3D). Several other genes that are also involved in stress response signaling and significantly induced in NGLY1-knockdown melanoma cells [16] were not upregulated in NGLY1-deficient COs. Despite the discrepancy between CO and melanoma cells in response to NGLY1 loss, NGLY1-deficient CO cells, compared with the functional ones, showed higher susceptibility to either bortezomib, glutamate, hydrogen peroxide, or thapsigargin (Fig. 4A–D). In contrast, they tolerated cisplatin treatment and glucose deprivation better than NGLY1-functional CO cells (Fig. 4E, F).

To test whether enhanced NGLY1 expression increases stress tolerability in developing cells, we expressed NGLY1 in cells isolated from EBs and COs of NGLY1-deficiency patient’s hiPSCs before pharmacologically stressing them. Expression of exogenous NGLY1 in the EB and CO cells attenuated bortezomib- and hydrogen peroxide−induced reduction of viability (Fig. 4G–I; Supplementary Fig. S5). Thus, altering NGLY1 activity could substantially affect the susceptibility and responses of developing human cells to stress.

### Premature differentiation of NSCs and perturbation of secreted factors discovered by single-cell analysis in NGLY1-deficient COs

**Fig. 5:**
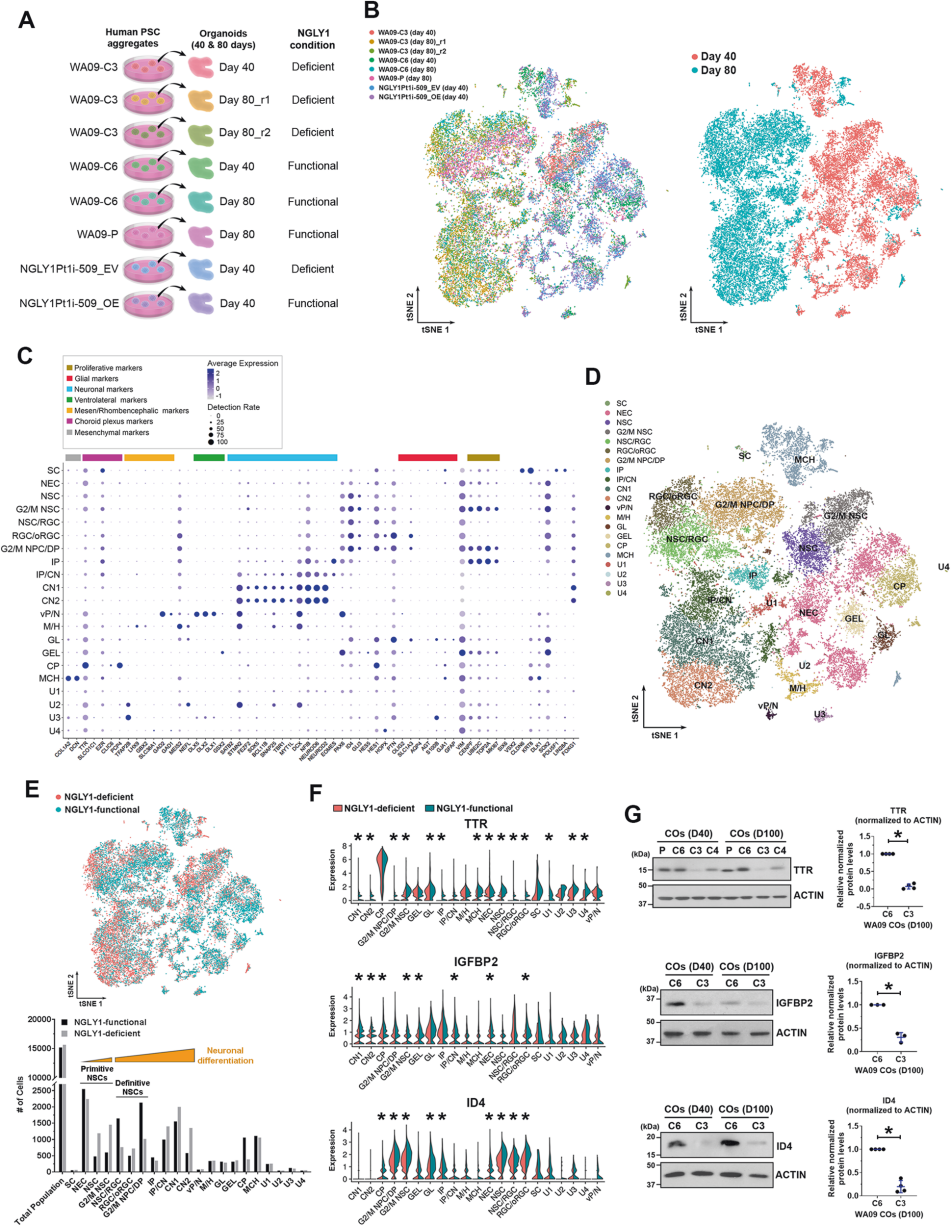
Single-cell RNA sequencing (scRNA-seq) analysis molecularly phenotypes cell populations, reveals composition changes, and identifies differentially expressed genes in NGLY1-functional and -deficient COs. **A** The schematic summary of different CO samples subjected to scRNA-seq analysis. **B** The tSNE plots of cells that were isolated from the indicated CO samples and subjected to scRNA-seq. *Left panel*: The tSNE plot of cells from each CO sample highlighted in a distinct color. *Right panel*: The tSNE plot of cells from day-40 and day-80 CO samples highlighted in distinctive colors. **C** Fourteen types of neural cells in the CO samples, together with choroid plexus and mesenchymal cells, were identified according to the expression profiles of 60 selected marker genes that are differentially expressed by various cell types associated with the developing brain. SC pluripotent stem cells, NEC neuroepithelial cells, NSC neural stem cells, G2/M NSC neural stem cells in G2/M phases, RGC radial glia, oRGC outer radial glia, G2/M NPC/DP: neuroprogenitors/dorsal progenitors in G2/M phases, IP: intermediate progenitors, CN1 and CN2: cortical neurons, vP/N ventral progenitors/neurons, M/H neuronal cells relevant to the midbrain/hindbrain, GL glial cells, GEL cells relevant to ganglionic eminence lateral, CP cells relevant to choroid plexus, MCH mesenchymal cells, U1–U4 unmapped cell types 1–4. **D** The tSNE plot of distinct cell types mapped using the expression profiles of the 60 selected marker genes. **E** *Top pane*l*:* The tSNE plot of cells from the NGLY1-functional and -deficient CO samples highlighted in distinctive colors. *Bottom panel:* The number of distinct cell type identified in the NGLY1-functional and -deficient CO samples. **F** The violin plots of TTR, IGFBP2, and ID4 expression across the identified cell types in NGLY1-functional and -deficient CO samples. *p < 0.05, MAST test. **G** The relative expression of TTR, IGFBP2, and ID4 detected by western blotting with densitometry analysis in NGLY1-functional and -deficient COs with 40 and 100 days of development. *P and C6:* COs developed from WA09-P and WA09-C6 (NGLY1-functional) hESCs. *C3 and C4:* COs developed from WA09-C3 and WA09-C4 (NGLY1-deficient) hESCs. *p < 0.05, *t*-test.

COs developed from NGLY1-functional and -deficient hESCs, as well as patient-derived hiPSCs with and without NGLY1 overexpression (Fig. 5A), were examined by scRNA-seq to molecularly phenotype cells at high resolution and determine cell types affected by NGLY1-deficient neurodevelopment. Each CO sample resulted in ~4500 analyzable single cells (Fig. 5B, left panel). Most day-80 CO cells showed transcriptomic features that separate them away from day-40 CO cells (Fig. 5B, right panel), suggesting that the cellular components of day-40 and -80 COs are fundamentally different due to developmental progression.

Based on the expression profiles of 60 marker genes (Fig. 5C), 14 neural cell types together with choroid plexus and mesenchymal cells in our COs were identified (Fig. 5D; Supplementary Fig. S6A, B). Given the negligible number of cells with hPSC features (Fig. 5E), cell differentiation was effective in COs. Day-40 COs largely consisted of primitive NSCs, choroid plexus, and mesenchymal cells, while day-80 COs were enriched by definitive NSCs, intermediate progenitor cells (IPCs), and cortical neurons (CNs) (Fig. 5B, D). Primitive NSCs with a higher degree of differentiation were found more in NGLY1-deficient COs compared with the functional ones (Fig. 5D, E). Similarly, NGLY1-deficient COs contained more differentiated CNs and fewer definitive NSCs (Fig. 5D, E). NGLY1-deficient COs at day 80, compared with the functional ones, also had higher abundances of TUBB3 and MAP2 proteins (Supplementary Fig. S7). These findings attest to NSC depletion and premature neuronal differentiation during neurogenesis without functional NGLY1.

*TTR* was the top downregulated gene in NGLY1-deficient COs (Fig. 5F). NGLY1 dysfunction in CO cells also hindered their expression of *IGFBP2* and *ID4* genes (Fig. 5F) that are involved in NSC maintenance during brain development [42–46]. Consistent with the RNA-seq results, TTR, IGFBP2, and ID4 proteins were reduced in NGLY1-deficient COs (Fig. 5G). In addition, HSPA8 and ISG15 expression that is associated with responses to multiple stress types [47, 48] was significantly changed in a few cell types of NGLY1-deficient COs (Supplementary Fig. S6C). Contrary to elevated ISG15 in NGLY1-deficiency patients’ lymphoblastoid cells [15], it was downregulated in human CO cells with dysfunctional NGLY1. After transplanting day-15 neuroepithelial spheres into the mouse cerebral cortex for an additional 50 days of in vivo development, NGLY1-deficient transplants hardly contained ID4-positive cells, in contrast to the functional counterparts with many ID4-positive cells (Supplementary Fig. S8). We also observed more RBFOX3-positive human neurons in the deficient transplants (Supplementary Fig. S8). Thus, perturbation of ID4 expression and neuronal differentiation due to NGLY1 dysfunction in COs can occur under a physiological condition and remain affected in a non-embryonic intracerebral environment with a normal NGLY1 ortholog in presence.

### Treatment with recombinant TTR and IGFBP2 enhances stress tolerability, NSC signaling, and proliferation that NGLY1 dysfunction attenuates in CO cells

**Fig. 6:**
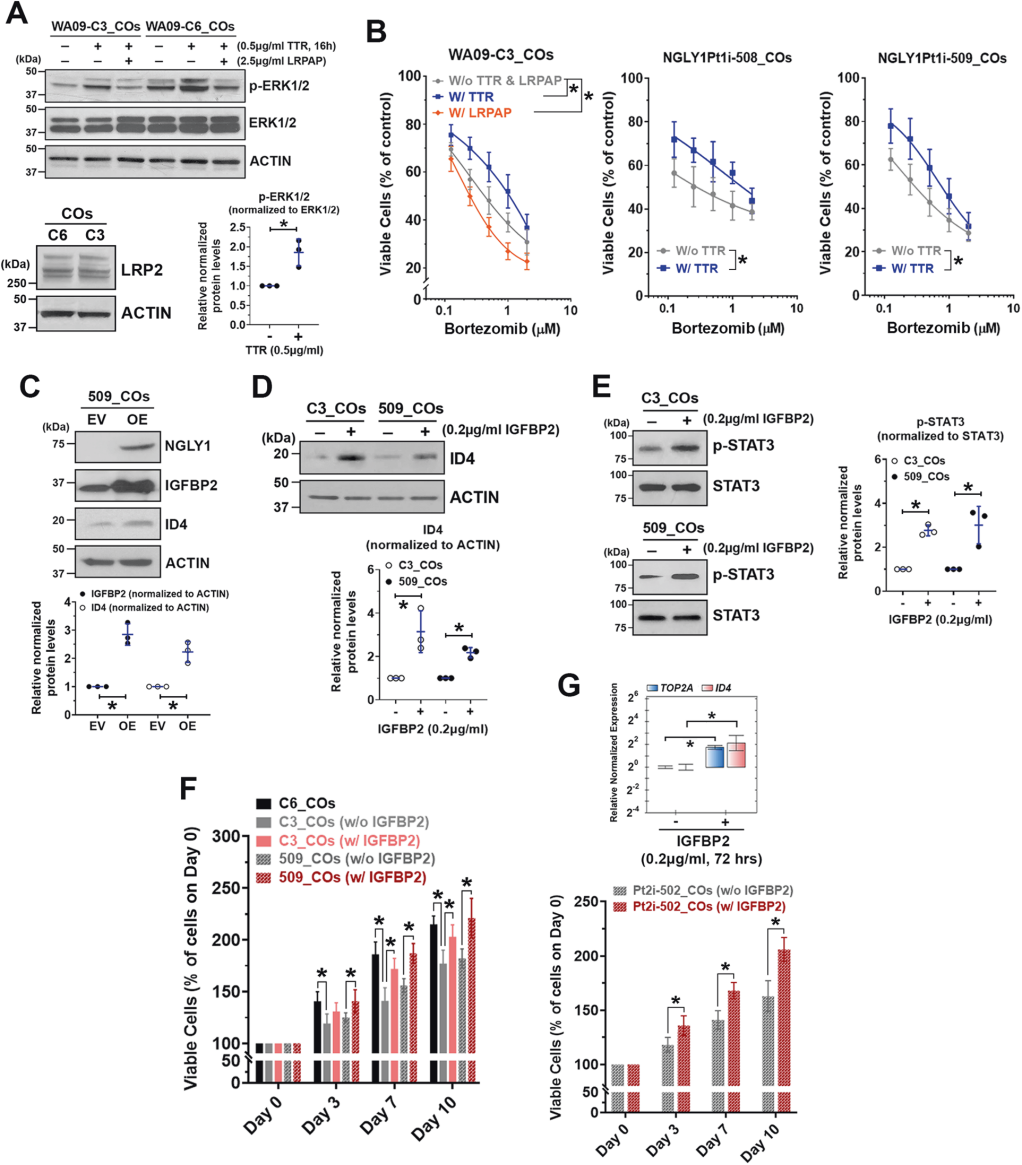
Supplementing recombinant TTR and IGFBP2 to NGLY1-deficient CO cells enhances their proteasome inhibitor tolerability, NSC-relevant signaling, and proliferation. COs collected at day 60 of development were dissociated to obtain CO cells. **A** *Top panel:* The expression and phosphorylation of ERK1/2 detected by western blotting in NGLY1-functional (WA09-C6) and -deficient (WA09-C3) CO cells with the indicated treatment of TTR and LDL receptor-related protein-associated protein 1 (LRPAP). The co-treatment of LRPAP, an LRP2 inhibitor, attenuated TTR-induced activation of MAPK signaling in the CO cells. *Bottom left panel:* The expression of LRP2 detected by western blotting in CO cells. *Bottom right panel:* The enhanced phosphorylation of ERK1/2 detected by western blotting with densitometry analysis in WA09-C3 CO cells that had TTR treatment for 16 h. *p < 0.05, *t*-test. **B** The viability of WA09-C3 CO cells in response to the indicated treatment of TTR, LRPAP, and bortezomib treatment was determined using MTT assays *(left panel*). *W/o TTR & LRPAP:* cells receiving bortezomib treatment without either TTR or LRPAP. *W/TTR:* cells receiving bortezomib treatment with the 4-hour pretreatment followed by 48-hour cotreatment of 0.5 µg/ml TTR. *W/LRPAP:* cells receiving bortezomib treatment with the concomitant treatment of 2.5 µg/ml LRPAP for 48 h. The viability of NGLY1Pt1i-508 and NGLY1Pt1i-509 CO cells in response to the indicated treatment of TTR and bortezomib was determined also using MTT assays (*middle and right panels*). *W/o TTR:* cells receiving bortezomib treatment without any TTR treatment. *W/TTR:* cells receiving bortezomib treatment with the 4-h pretreatment followed by 48-hour cotreatment of 0.5 µg/ml TTR. All data were presented as mean ± standard deviation (*n* = 3, *p < 0.05, logistic regression). **C** IGFBP2 and ID4 upregulation in response to the expression of exogenous NGLY1 detected by western blotting with densitometry in NGLY1Pt1i-509 CO cells. *EV:* Empty/control vector. *OE:* NGLY1 overexpression vector. *p < 0.05, *t*-test. **D** ID4 upregulation detected by western blotting with densitometry in WA09-C3 and NGLY1Pt1i-509 CO cells with IGFBP2 treatment. *p < 0.05, *t*-test. **E** The enhanced *p*hosphorylation of STAT3 detected by western blotting with densitometry in WA09-C3 and NGLY1Pt1i-509 CO cells with IGFBP2 treatment. *p < 0.05, *t*-test. **F** The proliferation of the indicated CO cells with and without the treatment of 0.2 µg/ml IGFBP2 for the indicated periods was determined using MTT assays. All data were presented as mean ± standard deviation (*n* = 3, *p < 0.05, *t*-test). **G** The TOP2A and ID4 expressio*n* (*top panel*) and prolifera*t*ion (*bottom panel*) of NGLY1Pt2i-502 CO cells with and without the treatment of 0.2 µg/ml IGFBP2 for the indicated periods were determined using qRT-PCR and MTT assays. All data were presented as mean ± standard deviation (*n* = 3, *p < 0.05, *t*-test).

By treating NGLY1-deficient CO cells with recombinant TTR and IGFBP2, we tested whether these secreted factors may alleviate NGLY1 deficiency-induced cellular abnormalities in neurogenesis. Because TTR can activate MAPK signaling through an LRP2 receptor-dependent mechanism and has been implicated in protecting stressed neurons [49], we examined ERK phosphorylation in CO cells with and without TTR treatment. Compared with NGLY1-functional CO cells, the deficient cells had reduced ERK phosphorylation (Fig. 6A, top panel). Treatment with TTR in both types of CO cells with similar LRP2 expression (Fig. 6A, bottom left panel) enhanced ERK phosphorylation (Fig. 6A, top and bottom right panels). TTR treatment-induced phosphorylation of ERK can be suppressed by co-treatment of recombinant LRPAP, an inhibitor for ligand binding to LDL receptor-related proteins (LRPs), in CO cells (Fig. 6A, *top panel*). While LRPAP treatment potentiated bortezomib toxicity in NGLY1-deficient CO cells, TTR treatment increased their tolerability (Fig. 6B).

The NGLY1 overexpression-induced concomitant upregulation of IGFBP2 and ID4 in NGLY1-deficiency patients’ CO cells (Fig. 6C; Supplementary Fig. S6D, E) indicated that ID4 expression modulated by NGLY1 may be coupled to IGFBP2 activity. Notably, treatment with recombinant IGFBP2 elevated ID4 in NGLY1-deficient CO cells (Fig. 6D). STAT3 phosphorylation was also enhanced in the NGLY1-deficient cells with IGFBP2 treatment (Fig. 6E). Moreover, patients’ CO cells showed ID4 upregulation and became more proliferative in response to IGFBP2 treatment (Fig. 6D, F, G). These findings suggest that NGLY1 may sustain the signaling and expansion of NSCs in the developing brain, at least partially, through maintaining IGFBP2 expression.

## Discussion

We delineated cellular and molecular features of NGLY1-defective human neurogenesis. In light of reduced upper-layer neurons (Fig. 2D) and attenuation of STAT3 and HES1 signaling critical for maintaining RGCs [39, 40] (Fig. 2E), NGLY1 malfunction could interfere with the functions of RGCs and other NSCs in human COs. Through bulk and single-cell transcriptomic analysis, we had direct evidence for the major impact of defective NGLY1 on neural precursors and their differentiation propensity (Figs. 3B and 5E). Considering the transcriptomic similarity between human COs and the fetal neocortex [18], NGLY1 loss−associated gene expression alterations in the COs likely show in the human NGLY1-dysfunctional fetal brain. Thus, NGLY1 deficiency could affect neural precursors and subsequently hinder the migration and development of upper-layer neurons in the human developing cerebrum. Similar to the enhanced propensity for neuronal differentiation we observed in NGLY1-deficient COs, another recent study that analyzed several cell types, including hiPSC-differentiated NPCs, from NGLY1-deficient patients also found gene expression relevant to neuron commitment and axonogenesis is significantly enriched in NGLY1-deficient NPCs compared to the functional ones [50].

Unlike melanoma cells, CO cells showed limited upregulation of ATF4 and GADD153 in response to NGLY1 loss (Fig. 3D), suggesting that NGLY1 dysfunction may cause distinct types or levels of stress in different cells. Although surprising, hyperactivation of stress response signaling does not appear a major cause of alterations in NGLY1-deficient human COs. Like our findings, wild-type and Ngly1-knockout rats have no detectable difference in the expression of ER stress markers [9]. Using various stressors (Fig. 4A–D), we demonstrated lower tolerability to proteotoxicity, excitotoxicity, oxidative stress, and ER stress in developing neural cells with dysfunctional NGLY1. However, those cells also appeared less sensitive to cisplatin and glucose deprivation (Fig. 4E, F). While additional studies are required to confirm our speculation, the higher tolerability to cisplatin and glucose starvation may be attributed to reduced cell proliferation because of premature differentiation in NGLY1-deficient COs. Concordant with the enhanced self-renewal in neural progenitors stimulated with hydrogen peroxide [51], treatment with low-dose hydrogen peroxide promoted proliferation of normal CO cells (Fig. 4C) that are composed of many progenitors. This proliferative response was abolished in NGLY1-defective CO cells (Fig. 4C), also suggesting that NGLY1 deficiency could substantially alter progenitor properties in the developing brain.

Our scRNA-seq analysis demonstrated NSC depletion accompanied by increased CNs due to NGLY1 loss in COs (Fig. 5C–E). Except for a few genes (Fig. 5F; Supplementary Fig. S6C), transcriptomic features in CNs with and without NGLY1 were largely similar. While understanding how NGLY1 loss may impact different CNs requires further investigation, gene expression relevant to vital activities in differentiated CNs seems mostly unaffected. Through the same analysis, significant downregulation of TTR due to NGLY1 loss in COs was identified (Fig. 5F, G). TTR makes up 25% of cerebrospinal fluid (CSF) protein [52]. Notably, most NGLY1-deficient children have total protein concentrations in their CSF below the normal range [6]. These findings indicate that TTR could be reduced and insufficient for normal development and function in the NGLY1-deficient brain. Besides facilitating thyroxin and retinol transportation in the body [53], TTR contributes to neuroprotection under ischemic stress via LRP2 binding and MAPK signaling activation [49, 54]. TTR also protects neurons from the proteotoxicity of amyloidogenic peptides [55–58]. While TTR reduction may also cause neural-specific hypothyroidism whereby prenatal and postnatal brain development can be stunted [59], our data from TTR and LRPAP treatment in NGLY1-deficient CO cells exposed to bortezomib (Fig. 6A, B) indicated that TTR downregulation due to NGLY1 malfunction could impair LRP2-mediated neuroprotection and subsequently affect stress tolerability in developing neural cells.

In addition to TTR, IGFBP2 and ID4 were highly reduced in NGLY1-deficient COs (Fig. 5F, G). NGLY1 loss also disturbed the kinetics of IGFBP2 and ID4 expression (Supplementary Fig. S9). Because IGFBP2 and ID4 maintain NSCs as well as regulate cerebral and cognitive development [44–46, 60], IGFBP2 and ID4 dysregulation during NGLY1-defective cortical development could limit proliferation and cause premature differentiation in NSCs, which may underlie microcephaly in NGLY1-deficiency patients. Notably, robust expression of ID4 in the human fetal brain starts from Carnegie stage 13 and persists during gestational weeks 6–10 [61]. Knowing ID4 downregulation in NGLY1-defective COs, the expression pattern of ID4 in the human fetal brain could be unsustainable under NGLY1 deficiency. Among four *ID* genes expressed in human cells, the expression of *ID4*, but not others, noticeably differed in response to NGLY1 loss (Supplementary Fig. S9). Thus, although all *ID* genes contribute to regulating embryogenesis [62, 63], NGLY1 deficiency appears to affect cerebral development particularly through disrupting ID4 function. With IGFBP2 treatment, NGLY1-deficient CO cells showed elevated ID4 expression, STAT3 phosphorylation, and proliferation (Fig. 6D–G). Aside from modulating insulin-like growth factors, IGFBP2 can activate MAPK and STAT3 signaling through integrin β1- and EGFR-dependent mechanisms [64, 65]. Collectively, IGFBP2 dysregulation could be a major cause of the molecular and phenotypic changes in NGLY1-deficient NSCs and underlie patients’ neurological defects.

NGLY1-mediated deglycosylation is known to facilitate retrotranslocation and degradation of misfolded BMP4, thereby allowing properly-folded BMP4 to proceed through the secretory pathway and activate signaling [66]. Ngly1-mutant mouse embryos also present a severe decrease in BMP signaling in certain contexts [66]. Our discoveries on TTR and IGFBP2 downregulation together with the reported dysregulation of BMP4 in NGLY1-defective cells suggest that NGLY1 deficiency could perturb many secreted factors needed for normal development. Of note, ID4 mediates the BMP4-induced inhibition of neuroprogenitor differentiation and lineage commitment [67, 68]. Because BMPs play a critical role in development and patterning of the central nervous system [69], whether BMP4 disruption is linked to ID4 downregulation in NGLY1-deficient cells and also contributes to abnormal cerebral development should be tested in the future.

Interestingly, expression of NGLY1 without deglycosylation activity appears to rescue the Ngly1-dependent function of transcriptional factors Creb1 and Atf1 in Ngly1-knockout mouse fibroblasts [70]. These data suggest NGLY1 could be capable of transcriptional regulation that is independent of its deglycosylation function. Given many transcription factors that are specifically expressed during neurogenesis and modulate NSC behavior, future work to delineate if the deglycosylation-independent action of NGLY1 affects transcriptional regulation in NSCs may lead to novel and important discoveries about NGLY1 in the context of neurodevelopment.

Overall, our study demonstrates the significant role of NGLY1 during neurogenesis in human COs, offers mechanistic insights into NGLY1-deficiency patients’ neurodevelopmental abnormalities, and reveals potential targets that we can further investigate to ultimately alleviate the disease phenotypes.

### Reporting summary

Further information on research design is available in the Nature Research Reporting Summary linked to this article.

## Supplementary information

Supplemental Methods and Data

Video S1_WA09-C6 CO

Video S2_WA09-C3 CO

Reporting Summary

## Data Availability

The microarray and scRNA-seq data have been deposited in the Gene Expression Omnibus (GEO) with accession numbers GSE169696 and GSE142143, respectively. The mass spectrometry data for proteomics analysis have been deposited in the ProteomeXchange with an identifier PXD026681.
