# Supplementary material for: Deficiency of N-glycanase 1 perturbs neurogenesis and cerebral development modeled by human organoids
Source: Cell Death Dis. 2022 Mar 24;13(3):262. doi: 10.1038/s41419-022-04693-0 (PMC8942998; doi:10.1038/s41419-022-04693-0)

Deficiency of *N*-glycanase 1 perturbs neurogenesis and cerebral development modeled by human organoids

Victor J.T. Lin[1†], Jiangnan Hu[1,2†], Ashwini Zolekar[1], Max R. Salick[3], Parul Mittal[4], Jordan T. Bird[5], Peter Hoffmann[4], Ajamete Kaykas[3], Stephanie D. Byrum[5], Yu-Chieh Wang[1,2]*

[1] Department of Pharmaceutical Sciences, UNT System College of Pharmacy, University of North Texas Health Science Center, Fort Worth, TX, USA

[2] Department of Dermatology, Medical College of Wisconsin, Milwaukee, WI, USA

[3] Department of Neuroscience, Novartis Institutes for Biomedical Research, Cambridge, MA, USA

[4] Future Industries Institute, University of South Australia, Adelaide, Australia

[5] Department of Biochemistry and Molecular Biology, University of Arkansas for Medical Sciences, Little Rock, AR, USA

*To whom correspondence should be addressed:

Yu-Chieh Wang, Ph.D.
Department of Dermatology
Medical College of Wisconsin
8701 Watertown Plank Rd., TBRC-C3885
Milwaukee, WI 53226
Tel: 1-(414) 955-2802
Fax: 1-(414) 955-0058
Email: yucwang@mcw.edu

† V.J.T.L. and J.H. equally contributed to this work.

Running title: NGLY1 malfunction in developing neural cells

Keywords: NGLY1, congenital deglycosylation disorder, neurodevelopment, neuroprogenitors, cerebral organoids, stress responses, secretory factors

Supplementary Information:

Materials and Methods

Whole-exome sequencing

A total amount of 1.0 μg genomic DNA per sample was used as input material for DNA library preparation. Sequencing libraries were generated using Agilent SureSelect human all exon kit (Agilent Technologies, CA) according to manufacturer's protocols, and index codes were added to each sample. Briefly, fragmentation was carried out by a hydrodynamic shearing system (Covaris, MA) to generate 180-280bp fragments. After adenylation of 3' ends of DNA fragments, adapter oligonucleotides were ligated. DNA fragments with ligated adapter molecules on both ends were selectively enriched in a PCR reaction. After PCR reaction, the library was hybridized with biotin-labeled probes, after which streptomycin-coated magnetic beads are used to capture the exons of genes. Captured libraries were enriched in a PCR reaction to add index tags to prepare for hybridization. Products were purified using AMPure XP system (Beckman Coulter, CA) and quantified using the Agilent high sensitivity DNA assay on a bioanalyzer 2100 system (Agilent Technologies, CA). Each library was sequenced on a HiSeq 4000 sequencer (Illumina, San Diego, CA). A quality control step was performed on raw sequencing data to filter adapter contaminated and low-quality reads (a read pair with more than 10% of bases uncertain in either one read or a read pair with a proportion of low-quality bases over 50% in either one read). Burrows-Wheeler Aligner (BWA) was utilized to map the paired-end clean reads to the human reference genome (b37+decoy, ftp://gsapubftp-anonymous@ftp.broadinstitute.org/bundle/b37/human_g1k_v37_decoy.fasta.gz). The original mapping results in a BAM format can be obtained. SAMtools was used for sorting the BAM files; Picard was used for annotating duplicate reads. Final BAM files were obtained to compute the read coverage and depth. Single nucleotide polymorphisms (SNPs) present in the library of each sample were detected by the analysis pipeline at Novogene (Sacramento, CA). Cluster analysis among the samples based on the detected SNPs was also performed.

Embryoid body (EB) formation

Undifferentiated hPSC aggregates generated using the AggreWell plates (Stemcell Technologies, Vancouver, Canada) were cultured in ultra-low attachment plates with DMEM/F12 medium with L-glutamine containing 20% KnockOut serum replacement, 100 μM non-essential amino

acids, and 100 μM β-mercaptoethanol (all from Thermo Fisher Scientific) for 7 days. The medium was changed every other day. On day 8, EBs developed from the hPSC aggregates were transferred into DMEM/F12 medium with L-glutamine containing 10% fetal bovine serum (Thermo Fisher Scientific, Carlsbad, CA) and cultured in the same medium for an additional 7 days.

Cerebral organoid (CO) formation

<u>hPSC medium:</u>

The hPSC medium consisted of 20% KnockOut serum replacement, 1% non-essential amino acids (NEAA), 1% GlutaMax, 0.18% 2-mercaptoethanol, 25 *n*g/ml FGF2 in DMEM/F12. All the components were obtained from Thermo Fisher Scientific (Carlsbad, CA).

<u>Neural induction medium:</u>

Neural induction medium containing SB431542 and IWR-1 was composed of 10 µM SB431542 (Stemcell Technologies, Vancouver, Canada) and 3 µM *endo*-IWR-1 (Stemcell Technologies, Vancouver, Canada), 1% N2 supplement, and 1 µg/ml heparin in the FGF2-deficient hPSC medium.

<u>Differentiation medium I, II and III:</u>

Differentiation medium I contained 1% N2 supplement, 0.025% insulin, 1% GlutaMax, 1% NEAA, penicillin-streptomycin (200 units/ml-200 µg/ml), 0.05% 2-mercaptoethanol in the Neurobasal™ medium. Differentiation medium II contained 1% N2 supplement, 2% B27 supplement without vitamin A, 0.025% insulin, 1% GlutaMax, 1% NEAA, penicillin-streptomycin (200 units/ml-200 µg/ml), 0.05% 2-mercaptoethanol in the Neurobasal™ medium. Differentiation medium III contained 1% N2 supplement, 2% B27 supplement with vitamin A, 0.025% insulin, 1% GlutaMax, 1% NEAA, penicillin-streptomycin (200 units/ml-200 µg/ml), 0.05% 2-mercaptoethanol in the Neurobasal™ medium. All the components of the differentiation media were obtained from Thermo Fisher Scientific (Carlsbad, CA).

The protocol for CO development was depicted in **Fig. 2a**. Growth factor-reduced Matrigel (Corning, Corning, NY) was used for embedding neuroepithelial spheres.

Immunofluorescence staining

For the staining of pluripotency biomarkers in hPSCs and their differentiated derivatives, cells were plated into 24-well plates, fixed and permeabilized, and incubated with primary antibodies against specific pluripotency biomarkers and fluorophore-conjugated secondary antibodies (Thermo Fisher Scientific, Carlsbad, CA). Organoid samples were fixed using 4% paraformaldehyde in PBS at 4°C overnight, rinsed with PBS, transferred into 15% sucrose in PBS at 4°C for 48 hours, and transferred subsequently into 30% sucrose in PBS at 4°C for 48 hours. The samples were frozen embedding in a tissue-freezing matrix (O.C.T. compound; Thermo Fisher Scientific, Carlsbad, CA) at -80°C. For the staining of frozen sections of organoid and tissue samples, sections on slides were rinsed in phosphate-buffered saline (PBS) three times to remove the tissue-freezing matrix, blocked with 5% donkey serum in PBS, and reacted with a primary antibody against a specific antigen at 4°C for overnight. After thorough washing with PBS containing 0.2% Tween-20 (PBST; Millipore Sigma, St. Louis, MO), the tissue sections were reacted with a fluorescence-conjugated secondary antibody (Thermo Fisher Scientific, Carlsbad, CA) at room temperature for 1 hour, washed with PBST, and counterstained using a DAPI solution. If the biotinylated primary antibody was used, fluorescence-conjugated streptavidin (Thermo Fisher Scientific, Carlsbad, CA) was subsequently used to react with the section. The stained organoid and tissue sections were mounted with coverslips using a Fluoromount-G medium (Southern Biotech, Birmingham, AL).

Optical clearing, staining and imaging in COs

CO samples were fixed in phosphate-buffered solution (PBS) containing 4% paraformaldehyde at room temperature for 30 minutes then immersed in the Scale CUBIC-1 solution made of 25% urea, 25% N,N,N’,N’-tetrakis(2-hydroxypropyl) ethylenediamine (THEED), and 25% Triton X in deionized water. Incubation occurred at 37°C with gentle shaking for 2 days. After clearing, the samples were washed in PBS at room temperature for 30 minutes and subsequently blocked in PBS containing 5% donkey serum overnight with gentle shaking at 37°C for 2 days. After blocking, PBS wash was performed at room temperature for 30 minutes before the primary antibodies against a specific antigen were applied to the samples at 37°C with gentle shaking for an additional 2-3 days. After thorough washing with PBS at room temperature for 30 minutes, fluorescence-conjugated secondary antibodies were applied for another 2 days at 37°C with gentle shaking. At the end of the reaction with secondary antibodies, thorough washing with PBS was performed at room temperature for 30 minutes. Before being imaged, COs were

submerged in a refractive index matching (RIM) solution made of 0.02M phosphate buffer, 40g Histodenz (Millipore Sigma, St. Louis, MO), 0.1% Tween 20, and 0.01% sodium azide. Once COs were fully clear, they were placed in a glass tube for being sectioned optically and imaged from both the left and right sides of each sample by a light sheet microscope (Zeiss, White Plains, NY). Dual side fusion based on max intensity matching for each stack was then applied followed by LUT normalization. The image of each focal plane in a CO sample was viewed with arivis Vision4D. The segmentation algorithm was built on a set of training images using the machine learning trainer in the software and subsequently applied to all the images in the analysis pipeline to enable the automated segmentation and enumeration of positively-stained cell nuclei in all the CO samples for each marker.

Cell viability test

EBs and COs were dissociated using a papain-based embryoid body dissociation kit (Miltenyi Biotec, San Diego, CA) to harvest viable cells. Cells were seeded into 96-well plates (3,000-8,000 cells/well, depending on cell types), incubated overnight, and treated as indicated. For the dissociated EB cells, 96-well plates were coated using growth factor-reduced Matrigel (Corning, Corning, NY) before cell seeding. For the dissociated CO cells, 96-well plates were coated using poly-L-ornithine (10 µg/ml; Advanced BioMatrix, Carlsbad, CA) followed by recombinant human laminin 521 (2.5 µg/ml; Thermo Fisher Scientific, Carlsbad, CA) before cell seeding. If DMSO was used as a vehicle to dissolve compounds and generate stock compound solutions for drug treatment, control groups received DMSO (0.1%, final concentration). After treatment, cells were incubated in FBS-free medium containing 0.4 mg/mL MTT (3-[4,5-dimethyl- thiazol-2-yl]-2,5-diphenyl-2H-tetrazolium bromide; TCI America, Portland, OR) at 37°C for 1 hour. Reduced MTT was solubilized in DMSO for measuring absorbance at 570 nm. The relative cell viability in each treatment condition was calculated based on absorbance values. For determining the percentages of apoptotic cells, cell samples (~1 x 10^6 cells per sample) stained with Annexin V-Alexa Fluor 647 (Thermo Fisher Scientific, Carlsbad, CA) according to the manufacturer's instruction were analyzed using an LSR II flow cytometer (BD Biosciences, Franklin Lakes, NJ).

Gene expression analysis by microarrays and qRT-PCR

Total RNA was isolated from cell samples using the mirVana miRNA Isolation Kit (Thermo Fisher Scientific, Carlsbad, CA). The quality of each RNA sample was determined using an

Agilent 2200 Tape Station system (Agilent, Santa Clara, CA) for RNA integrity analysis. Samples with RIN^e numbers above 7 were chosen to move forward with global gene expression profiling. Global gene expression profiling was performed using HT-12v4 Human Gene Expression Bead Chips and an iScan array scanning system (Illumina, Hayward, CA), according to the manufacturer's instructions. The gene expression array data have been deposited with a link to an accession number GSE169696 in the Gene Expression Omnibus (GEO). Data were filtered for detection *p*-value <0.01 in GenomeStudio (Illumina, Hayward, CA), and normalized using the LUMI package with RSN (Robust spline normalization) algorithm in R. The limma package in R was used for multivariate analysis to identify the top differentially expressed genes ($p<0.05$). The volcano plots were obtained using the limma package in conjunction with the ggplot2 package in R. Multiplex qRT-PCR was performed using cDNA generated from the RNA samples and Taqman® assays for the *SRY, IGFBP2, ID4, TOP2A* and *ACTB* (internal control) genes (assay ID# Hs00976796_s1, Hs01040719_m1, Hs02912975_g1, Hs01032137_m1 and Hs03023943_g1; Thermo Fisher Scientific, Carlsbad, CA), according to the manufacturer's instructions.

Single-cell RNA sequencing

Preparation and sequencing of cDNA library:

The chromium single-cell 3′ kit (v2) from 10x Genomics (Pleasanton, CA) was used to enable cDNA preparation in each cell suspension on the chromium controller (10x Genomics, Pleasanton, CA) according to the manufacturer's protocol. A cDNA amplification reaction mixture containing the amplification master mix (10x Genomics, Pleasanton, CA), cDNA additive (10x Genomics, Pleasanton, CA), cDNA primer mix (10x Genomics, Pleasanton, CA), and nuclease-free water was used to amplify cDNA in 12 amplification cycles. SPRIselect beads (Beckman Coulter, Indianapolis, IN) were used to isolate cDNA. The yield of cDNA was measured on a 2100 bioanalyzer (Agilent, Santa Clara, CA). The cDNA samples after enzymatic fragmentation, end repair, and A-tailing using a reaction mixture containing fragmentation enzyme blend in a buffer (10x Genomics, Pleasanton, CA) were then purified using the SPRIselect beads, before performing adaptor ligation and indexing PCR with the relevant reagents acquired from 10x Genomics (Pleasanton, CA). The indexed cDNA samples were sequenced on a HiSeq 4000 sequencer using HiSeq 3000/4000 SBS kits (Illumina, San Diego,

CA). The 10x Cell Ranger 1.3.1 pipeline was used to convert raw BCL files to cell-gene matrices. Briefly, the Illumina bcl2fastq script conducted the initial demultiplexing. FASTQ files were then aligned, UMI-filtered, and barcodes were matched via the *cellranger count* script. The grch37.75 human reference genome was used for alignment. After filtering out barcodes with very few matching transcripts, a total of ~34000 cells across 8 analyzed samples were adequately sequenced. Around 62000 post-normalization mean reads were detected in a cell, with ~2,194 as the median number of genes detected per cell. All the data were combined via the *cellranger aggr* script.

Data analysis:

The R package of Seurat was used to further analyze the filtered, aggregated, and depth normalized counts generated by *cellranger count* and *cellranger aggr* (http://software.10xgenomics.com/single-cell/overview/welcome).[1] Cells with unique feature counts more than the 75th percentile plus 1.5 times the interquartile range (IQR) or less 200 unique features, total gene counts more than the 75th percentile plus 1.5 times the IQR or less than 1000 counts, and/or mitochondrial feature percentage more the 75th percentile plus 1.5 times the IQR or less than the 25th percentile minus 1.5 times the IQR were filtered out of the data. Next, the 2000 highest variable features are selected. The data were then scaled by a linear transformation, where variation associated with cell death and cell cycling was regressed out of the scaled data using mitochondrial feature percentage and cell cycle scoring values. Principal component analysis (PCA) was performed on the scaled data. A JackStraw procedure was implemented to determine the significant PCA components that have a strong enrichment of low *p*-value features.

Clustering methods within Seurat were used to embed cells in a graph structure a K-nearest neighbor (KNN) graph, with edges drawn between cells with similar feature expression patterns into highly interconnected ‘quasi-cliques’ or ‘communities’. A t-distributed stochastic neighbor embedding (tSNE) was used to visualize and explore the results of this clustering. Seurat’s *FindNeighbors* and *FindClusters* functions were optimized to label clusters based on the visual clustering in the projections. Seurat *FindAllMarkers* function was used to call markers that define clusters by differential expression. It identified positive markers of a single cluster compared to all other cells and outputs the differential expression results. These markers will be

compared to known markers of expected cell types in order to assign appropriate cell type labels.[2] Differential expression analysis was performed using the *FindMarkers* to compare similar cell types across NGLY1-deficient and NGLY1-functional cerebral organoid samples. The MAST test, a GLM-framework that treats cellular detection rate as a covariate, was used to determine statistically different transcription.[3]

Proteomics analysis

Samples were processed using filter-aided sample preparation (FASP) protocol with slight modifications.[4] Briefly, the samples were solubilized in 8M urea, 1% (w/v) sodium dodecyl sulfate (SDS), 100 mM ammonium bicarbonate (AmBic), and 1% protease inhibitor cocktail (all from MilliporeSigma, Burlington, MA) and sonicated on ice for 5 minutes. Samples were reduced with 1M DTT and incubated at 30°C for 1 hour, followed by centrifugation at 13,000 RCF for 5 minutes. Around 50 µg of the reduced samples were transferred onto pre-rinsed Vivacon 500 spin column (30 kD MWCO; Sartorius, Goettingen, Germany), washed using 8 M urea in 100 mM AmBic, and spun for 10 minutes at 13,000 RCF at room temperature. SDS was removed by washing three times with 8M urea in 100 mM AmBic and spinning for 10 minutes at 13,000 RCF. Reduced samples were alkylated with 55 mM iodoacetamide (IAA) in 100 mM AmBic for 20 min in dark at room temperature. IAA was then removed by washing two times with 8M urea in 100 mM AmBic, and again two times with 50mM AmBic in a similar fashion. Five µg of trypsin (sequencing grade, Promega) in 10 mM AmBic was added and incubated rocking overnight at 37°C. Following overnight digestion, samples were centrifuged at 13,000 RCF and the flow-through was collected, reduced to ~1 µL by vacuum centrifugation then resuspended in 3% acetonitrile (CAN). The peptide concentration was measured on a NanoDrop 2000/2000c spectrophotometer (Thermo Fisher Scientific, Carlsbad, CA) at 205 nm wavelength. Samples were then acidified to a final concentration of 0.1% (v/v) trifluoroacetic acid.

The LC-MS/MS of digested proteins was performed using an Ultimate 3000 nano-flow system (Thermo Fisher Scientific, Carlsbad, CA) coupled to an LTQ XL Orbitrap ETD MS instrument (Thermo Fisher Scientific, Carlsbad, CA). Around 1.5 µg of peptide samples were pre-concentrated onto a C18 trapping column (Acclaim PepMap100 C18 75 µm × 20 mm; Thermo-Fisher Scientific, Carlsbad, CA) for 10 minutes at a flow rate of 5 µL/min, buffer-used 2% acetonitrile in 0.1% formic acid. Peptides were then separated at a flow rate of 300 nL/min by a

15 cm long C18 separation column (Acclaim PepMap100 C18 75 μm × 15 cm; Thermo-Fisher Scientific, Carlsbad, CA) with a linear gradient of 5% – 45% buffer B for 65 munites, followed by a 5-minute wash with 90% buffer B, and a 15-minute equilibration with 5% buffer B (buffer A: 2% acetonitrile in 0.1% formic acid, buffer B: 80% acetonitrile in 0.1% formic acid). LC and MS acquisition was controlled by Xcalibur version 2.1 (Thermo Fisher Scientific, Carlsbad, CA). The LTQ-Orbitrap instrument was operated at positive ion mode with normalized collision energy set to 35%. All MS and MS/MS spectra were acquired in the data-dependent mode in the full mass range of *m/z* 300 to 2000 at a resolution of 60000 in the FT mode. Ten most intense precursor ions were selected for MS/MS scan by collision-induced dissociation (CID) using a dynamic exclusion of 5 seconds, the minimum relative signal intensity of 1000, and a ≥2 positive charge state. All samples were performed in technical triplicate analysis.

To identify and quantify the protein peptides, MS/MS spectra were analyzed and subjected to label-free quantification (LFQ) using MaxQuant software (version 1.5.2.8) with the Andromeda search engine against the Uniprot human database. Search parameters used were a maximum number of miss-cleavages for trypsin as two per peptide. Carbamidomethylation of cysteine was set as a fixed modification. Methionine oxidation was considered as a variable modification. LFQ was activated with a minimum ratio count of 2 as well as allowed matching between runs and unidentified features. The standard Orbitrap settings were used with the mass tolerances for MS and MS/MS were 20 ppm and 0.5 Da. Only proteins that were identified with more than 2 peptides with a minimum length of 7 amino acids were considered for further analysis.

Three technical replicates for each sample in each developmental time point were evaluated and statistically calculated using the Perseus software (version 1.6.0.2). MaxQuant data was filtered for reverse identifications, contaminants, and “only identified by site". The LFQ intensities were log-transformed and imputed using the average minimum log intensity across runs. Based on MS intensity, the average percentage for each protein in total peptides detected in each replicate was calculated. The abundance ratio of each identified protein was calculated using their average percentages in NGLY1-functional and -deficient samples.

In vivo Studies

Eight-week-old female NCG (NOD-*Prkdc*^{em26Cd52}*Il2rg*^{em26Cd22}/NjuCrl) mice (Charles River, Wilmington, MA) were group-housed under conditions of constant photoperiod (12 hours light:

12 hours dark) with *ad libitum* access to sterilized food and water. COs developed from WA09-C6 and WA09-C3 hESCs were used in the animal study. The number of animals included in this study was chosen based on our prior experience from another similar study on transplanted COs. Each isoflurane-anesthetized mouse was placed stably in a stereotactic frame with its body temperature maintained at 37 °C using a heating pad. Small incisions were made at the skin above the skull that covers the mouse cerebral cortex. Craniotomy was performed to create a circular hole of ~3mm diameter through the skull above a transplantation site. Subsequently, the meninges underneath the hole was removed to access the brain and create a small cavity in the cortex by aspiration with a blunt-end needle to house cerebral organoids. The cavity was created unilaterally at the retrosplenial cortex. COs without Matrigel embedding at day 15 of development from the same batch of organoid production were inserted into the cavity. Each transplantation site received 3 organoids of similar size. The transplanted region was covered with a 3-mm coverslip that was mounted to the skull using adhesive glue to form a cranial window. The surgical wound was closed with sutures. After the surgery, the animals were allowed to recover in a temperature-controlled cage before returning to their regular cages. The general condition of the animals was monitored daily throughout the entire study.

At terminal sacrifice, mice were anesthetized with a lethal dose of isoflurane and perfused transcardially with PBS (pH 7.2–7.5) followed by PBS containing 4% paraformaldehyde (PFA) freshly prepared. The whole animal brain was harvested and further fixed in PBS containing 4% PFA overnight. After fixation, the brain was transferred into 30% sucrose in PBS for 48 hours and subsequently frozen embedding in the O.C.T. compound at -80°C before cryosection for immunofluorescence staining. Researchers who assessed the results were blinded to the information of group allocation during experiments and data analysis.

References Cited in Supplementary Materials and Methods

1 Butler A, Hoffman P, Smibert P, Papalexi E, Satija R. Integrating single-cell transcriptomic data across different conditions, technologies, and species. *Nature biotechnology* 2018; **36**:411-420.

2 Franzen O, Gan LM, Bjorkegren JLM. PanglaoDB: a web server for exploration of mouse and human single-cell RNA sequencing data. *Database (Oxford)* 2019; **2019**.

3 Finak G, McDavid A, Yajima M *et al.* MAST: a flexible statistical framework for assessing transcriptional changes and characterizing heterogeneity in single-cell RNA sequencing data. *Genome Biol* 2015; **16**:278.

4 Wisniewski JR, Zougman A, Nagaraj N, Mann M. Universal sample preparation method for proteome analysis. *Nature methods* 2009; **6**:359-362.

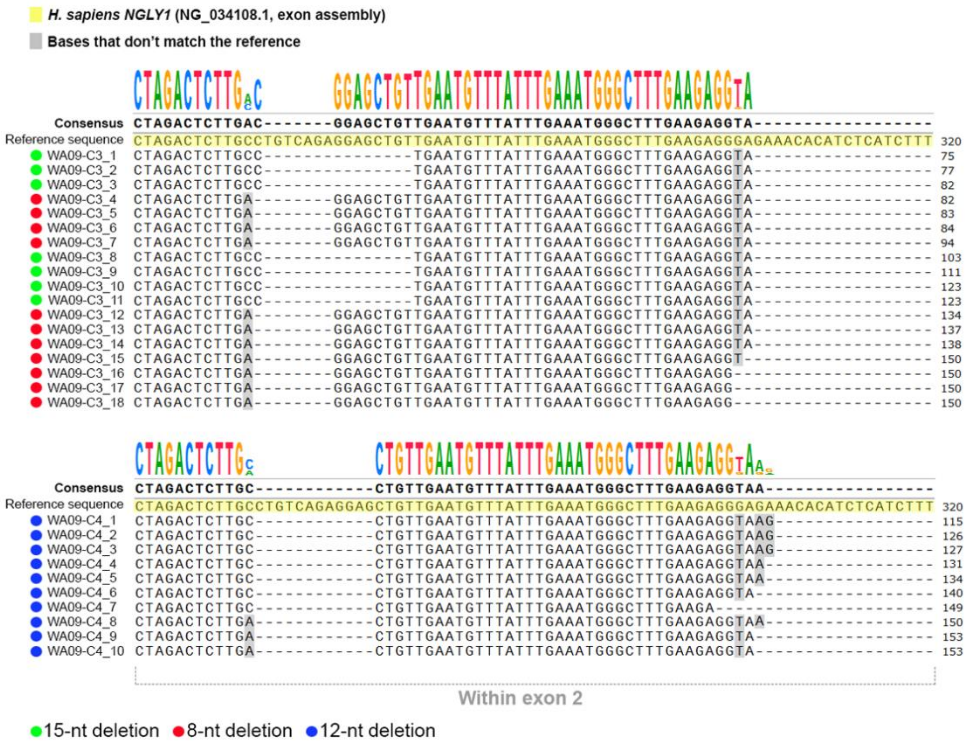

H. sapiens NGLY1 (NG_034108.1, exon assembly)
Bases that don't match the reference
Consensus
Reference sequence
Within exon 2
15-nt deletion
8-nt deletion
12-nt deletion

Fig. S1. Deletion mutations in NGLY1-deficient WA09 hESCs. Deletion mutations induced by CRISPR-Cas9-mediated editing in exon 2 of the *NGLY1* gene were confirmed by DNA sequencing in WA09-C3 and WA09-C4 hESCs. The PCR amplicons of the gene-editing site cloned into a TA cloning vector were sequenced.

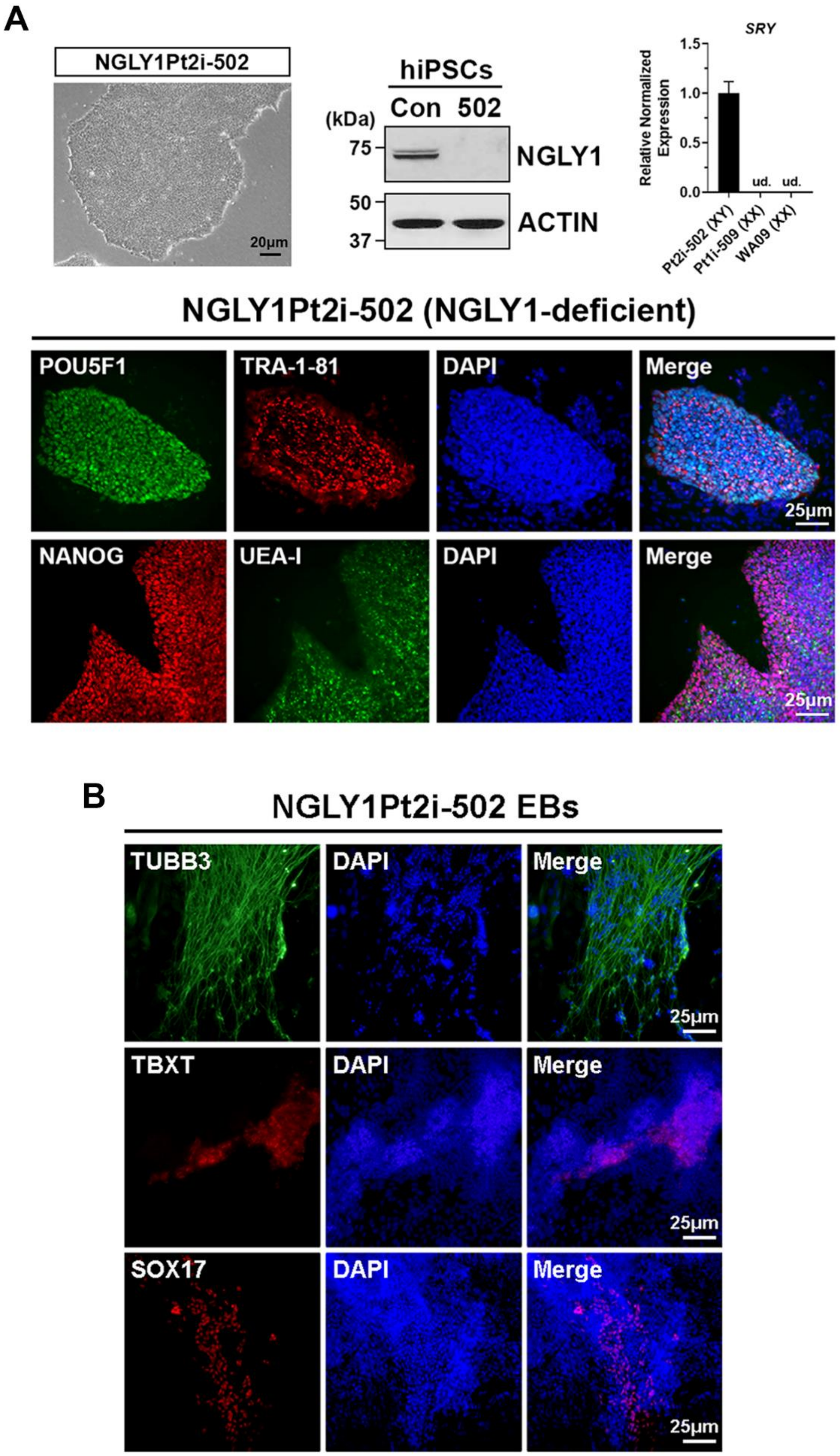
A
NGLY1Pt2i-502
20μm
hiPSCs
Con
502
(kDa)
75
NGLY1
50
37
ACTIN
SRY
Relative Normalized Expression
1.5
1.0
0.5
0.0
ud.
ud.
Pt2i-502 (XY)
Pt1i-509 (XX)
WA09 (XX)
NGLY1Pt2i-502 (NGLY1-deficient)
POU5F1
TRA-1-81
DAPI
Merge
25μm
NANOG
UEA-I
DAPI
Merge
25μm
B
NGLY1Pt2i-502 EBs
TUBB3
DAPI
Merge
25μm
TBXT
DAPI
Merge
25μm
SOX17
DAPI
Merge
25μm

Fig. S2. NGLY1-deficiency patient–derived hiPSCs are positively stained with pluripotency markers and capable of EB formation. (**A**) *Top left panel:* a typical hPSC morphology of NGLY1Pt2i-502 hiPSCs. *Top middle panel:* NGLY1 protein expression detected by western blotting in normal (Con) and NGLY1Pt2i-502 hiPSCs. *Top right panel:* The expression of the Y chromosome–specific *SRY* gene was detected by qRT-PCR in NGLY1Pt2i-502 hiPSCs but undetectable (ud.) in NGLY1Pt1i-509 hiPSCs and WA09 hESCs. *Bottom panel:* The staining of pluripotency markers, including TRA-1-81, UEA-I, POU5F1, and NANOG, in NGLY1Pt2i-502 hiPSCs. (**B**) EBs containing cells relevant to three germ-layer lineages were developed from NGLY1Pt2i-502 hiPSCs. *TUBB3:* an ectoderm marker. *TBXT:* a mesoderm marker. *SOX17:* an endoderm marker.

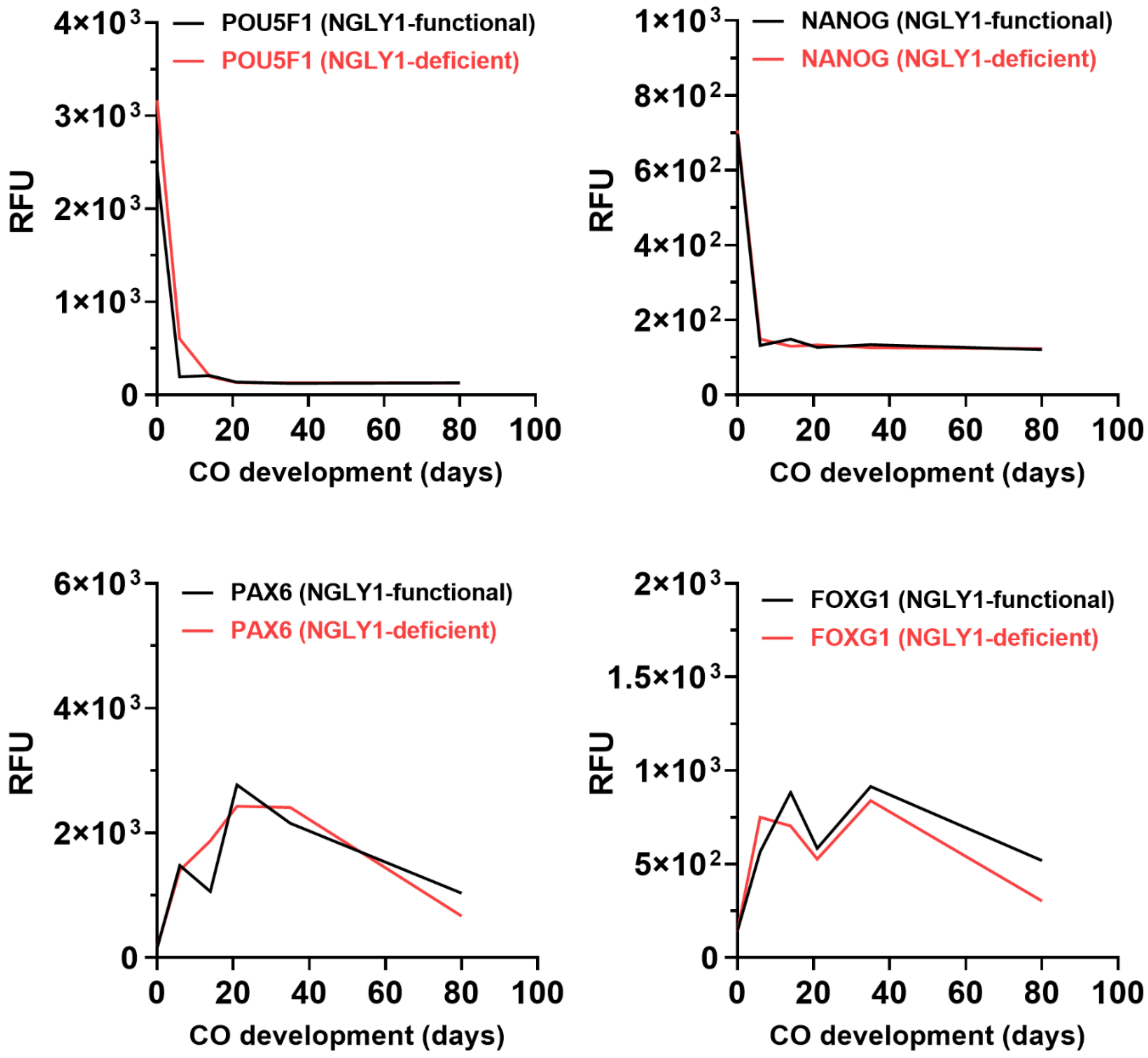

POU5F1 (NGLY1-functional)
POU5F1 (NGLY1-deficient)
RFU
4×10³
3×10³
2×10³
1×10³
0
0 20 40 60 80 100
CO development (days)
NANOG (NGLY1-functional)
NANOG (NGLY1-deficient)
RFU
1×10³
8×10²
6×10²
4×10²
2×10²
0
0 20 40 60 80 100
CO development (days)
PAX6 (NGLY1-functional)
PAX6 (NGLY1-deficient)
RFU
6×10³
4×10³
2×10³
0
0 20 40 60 80 100
CO development (days)
FOXG1 (NGLY1-functional)
FOXG1 (NGLY1-deficient)
RFU
2×10³
1.5×10³
1×10³
5×10²
0
0 20 40 60 80 100
CO development (days)

Fig. S3. The expression patterns of the *POU5F1, NANOG, PAX6* and *FOXG1* genes revealed by microarray analysis in NGLY1-functional and -deficient COs at different time points during 80 days of development. COs were developed from NGLY1-functional and -deficient WA09 hESCs. Samples were collected at either day 0, 6, 14, 21, 35, or 80 for microarray analysis. Average relative fluorescence units (RFUs, *n*=4 from each NGLY1 condition at each time point) associated with the indicated gene probes in the normalized array data were plotted along the course of CO development.

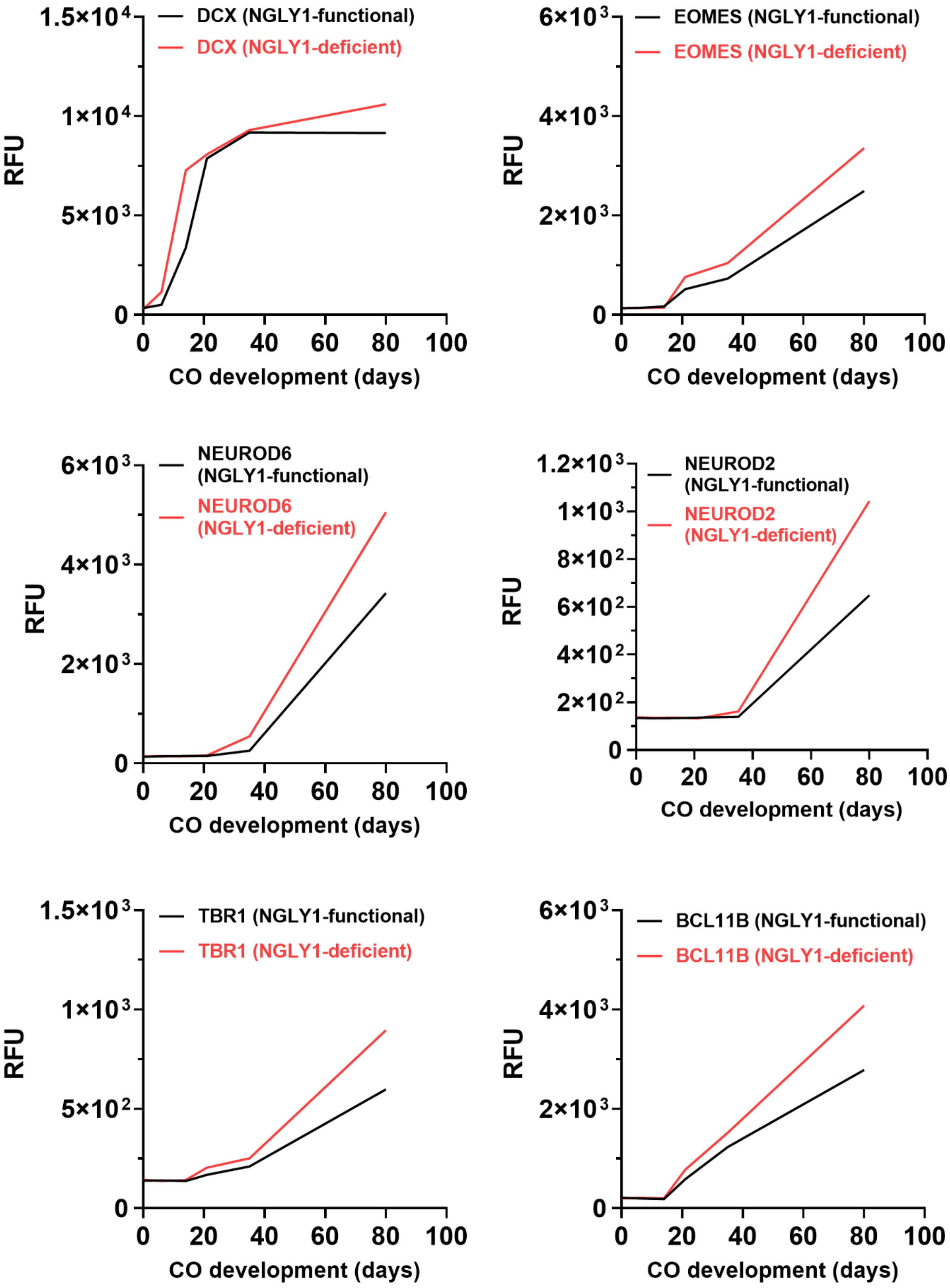
DCX (NGLY1-functional)
DCX (NGLY1-deficient)
EOMES (NGLY1-functional)
EOMES (NGLY1-deficient)
NEUROD6 (NGLY1-functional)
NEUROD6 (NGLY1-deficient)
NEUROD2 (NGLY1-functional)
NEUROD2 (NGLY1-deficient)
TBR1 (NGLY1-functional)
TBR1 (NGLY1-deficient)
BCL11B (NGLY1-functional)
BCL11B (NGLY1-deficient)
RFU
CO development (days)
0
20
40
60
80
100
1.5×10^4
1×10^4
5×10^3
6×10^3
4×10^3
2×10^3
1.2×10^3
1×10^3
8×10^2
6×10^2
4×10^2
2×10^2
1.5×10^3
5×10^2

Fig. S4. The expression patterns of the *DCX, EOMES, NEUROD6, NEUROD2, TBR1* and *BCL11B* genes revealed by microarray analysis in NGLY1-functional and -deficient COs at different time points during 80 days of development. COs were developed from NGLY1-functional and -deficient WA09 hESCs. Samples were collected at either day 0, 6, 14, 21, 35, or 80 for microarray analysis. Average relative fluorescence units (RFUs, *n*=4 from each NGLY1 condition at each time point) associated with the indicated gene probes in the normalized array data were plotted along the course of CO development.

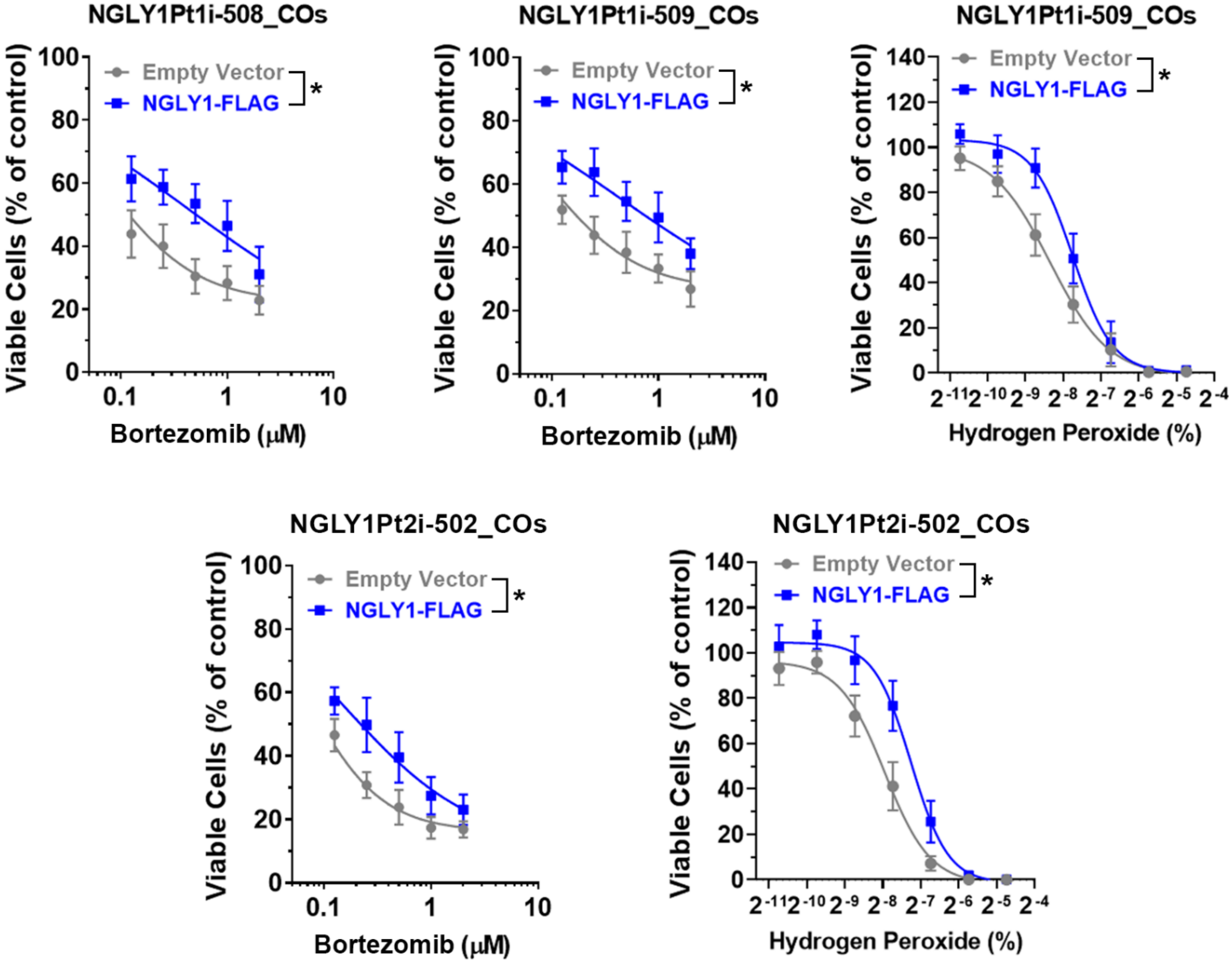
NGLY1Pt1i-508_COs
NGLY1Pt1i-509_COs
NGLY1Pt1i-509_COs
NGLY1Pt2i-502_COs
NGLY1Pt2i-502_COs
Empty Vector
NGLY1-FLAG
*
Viable Cells (% of control)
Bortezomib (μM)
Hydrogen Peroxide (%)
0.1
1
10
2^-11 2^-10 2^-9 2^-8 2^-7 2^-6 2^-5 2^-4

Fig. S5. The ectopic expression of NGLY1 in CO cells developed from NGLY1-deficiency patient–derived hiPSCs enhances their tolerability to bortezomib and hydrogen peroxide. COs collected at ~day 40 of development were dissociated to obtain CO cells. Cell viability was determined by MTT assays. The reduced susceptibility to the 48-hour treatment of bortezomib and the 24-hour treatment of hydrogen peroxide in NGLY1Pt1i-508, NGLY1Pt1i-509 and NGLY1Pt2i-502 CO cells with expression of exogenous NGLY1. All data were presented as mean ± standard deviation (n=3, *p<0.05, logistic regression).

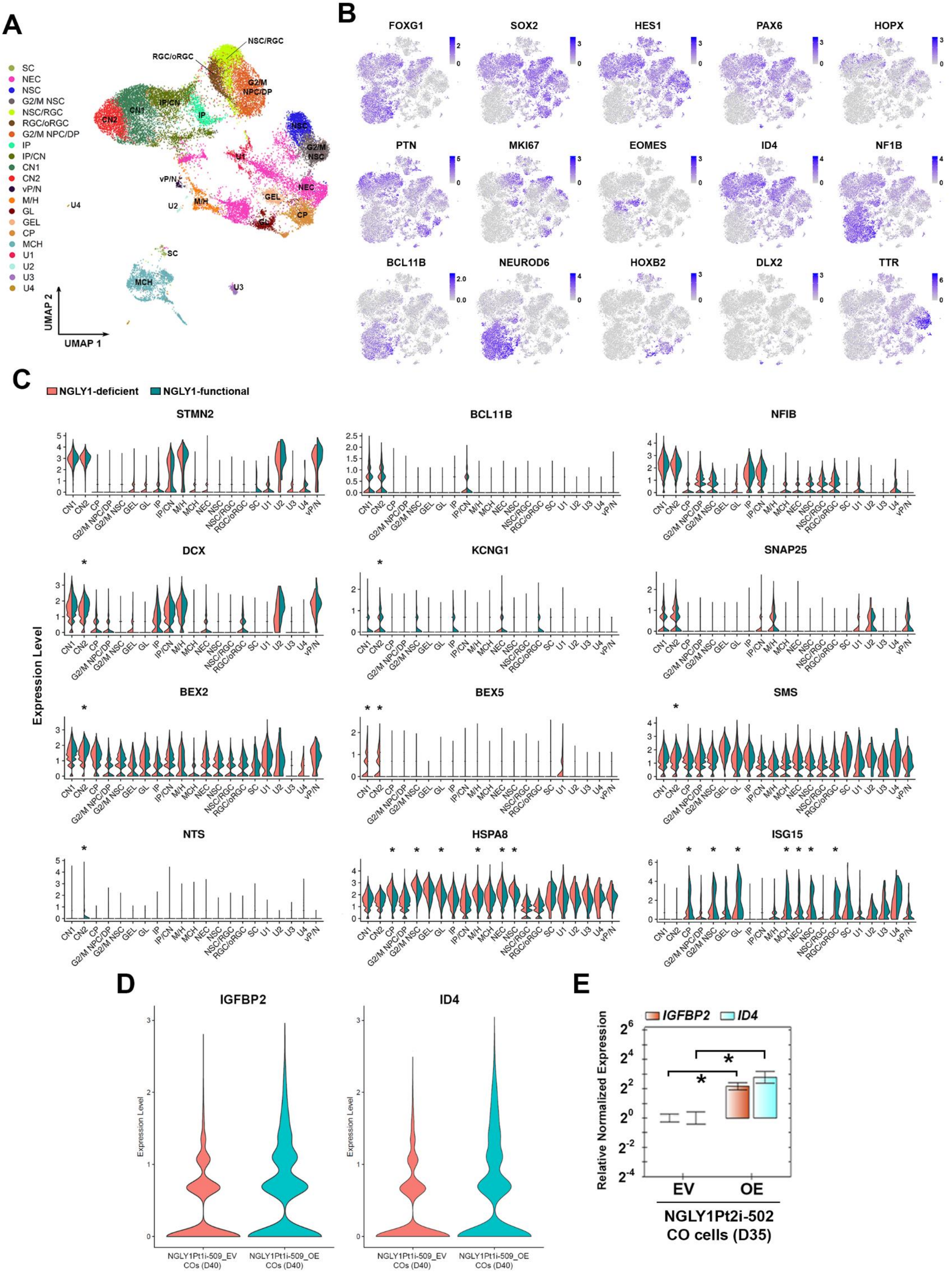
A
SC
NEC
NSC
G2/M NSC
NSC/RGC
RGC/oRGC
G2/M NPC/DP
IP
IP/CN
CN1
CN2
vP/N
M/H
GL
GEL
CP
MCH
U1
U2
U3
U4
UMAP 1
UMAP 2
B
FOXG1
SOX2
HES1
PAX6
HOPX
PTN
MKI67
EOMES
ID4
NF1B
BCL11B
NEUROD6
HOXB2
DLX2
TTR
C
NGLY1-deficient
NGLY1-functional
STMN2
BCL11B
NFIB
DCX
KCNG1
SNAP25
BEX2
BEX5
SMS
NTS
HSPA8
ISG15
Expression Level
D
IGFBP2
ID4
Expression Level
NGLY1Pt1i-509_EV COs (D40)
NGLY1Pt1i-509_OE COs (D40)
E
IGFBP2
ID4
Relative Normalized Expression
EV
OE
NGLY1Pt2i-502 CO cells (D35)

Fig. S6. The heterogeneity of cells developed in NGLY1 functional and -deficient COs. (**A**) The UMAP plot of 21 cell types identified through molecular phenotyping in scRNA-seq analysis. The proximity of the G2/M NPC/DP, NSC/RGC, and RGC/oRGC clusters that are bridged into the CN1 and CN2 clusters by the IP and IP/CN clusters indicate a differentiation trajectory from definitive NSCs to intermediate progenitors that are further matured into cortical neurons in both NGLY1-functional and -deficient COs. *SC:* pluripotent stem cells. *NEC:* neuroepithelial cells. *NSC:* neural stem cells. *G2/M NSC:* neural stem cells in G2/M phases. *RGC:* radial glia. *oRGC:* outer radial glia. *G2/M NPC/DP:* neuroprogenitors/dorsal progenitors in G2/M phases. *IP:* intermediate progenitors. *CN1 and CN2:* cortical neurons. *vP/N:* ventral progenitors/neurons. *M/H:* neuronal cells relevant to the midbrain/hindbrain. *GL:* glial cells. *GEL:* cells relevant to ganglionic eminence lateral. *CP:* cells relevant to choroid plexus. *MCH:* mesenchymal cells. *U1-U4:* unmapped cell types 1-4. (**B**) The *t*SNE plots with color based on the expression of representative marker genes used for molecular phenotyping. (**C**) The expression of selected marker genes relevant to neuronal cells and stress responses in NGLY1-functional and -deficient CO cells. (**D, E**) The enhanced expression of *IGFBP2* and *ID4* genes in patient-derived, NGLY1-deficient CO cells with the expression of exogenous NGLY1. Gene expression in NGLY1Pt2i-502 CO cells was analyzed by qRT-PCR (n=3 for each transduction vector, *$p<0.05$, t-test). *D40:* CO development for 40 days. *D35:* CO development for 35 days.

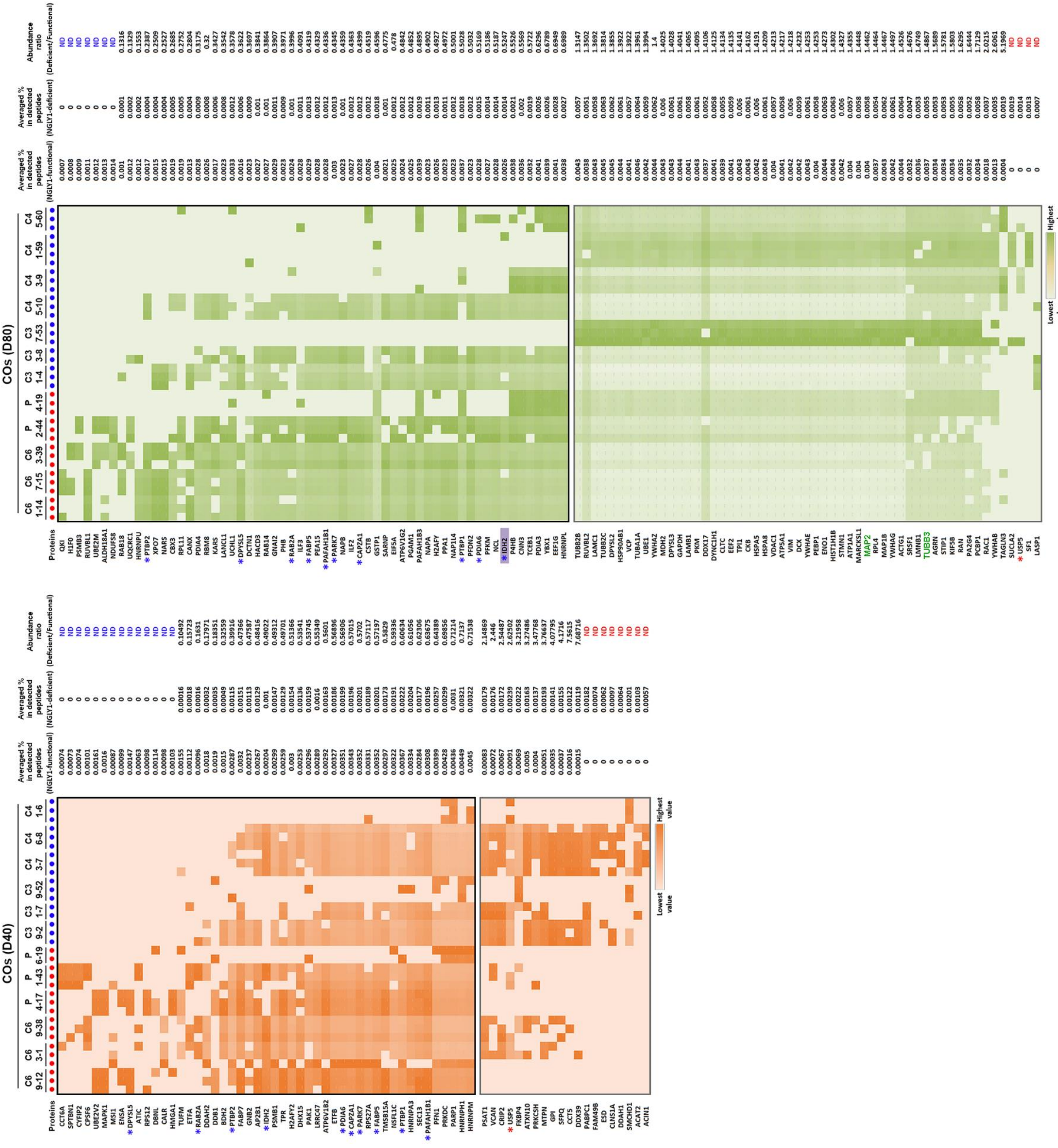
COs (D40)
COs (D80)
Proteins
Averaged % in detected peptides (NGLY1-functional)
Averaged % in detected peptides (NGLY1-deficient)
Abundance ratio (Deficient/Functional)
Lowest value
Highest value

Fig. S7. The NGLY1 deficiency-induced alterations of protein abundance detected by proteomics analysis in COs developed from NGLY1-functional and -deficient WA09 hESCs. The heatmap representations of differentially abundant proteins ($p < 0.05$, *t*-test) in the NGLY1-functional and -deficient COs with 40 and 80 days (D40 and D80) of development. The increased abundance of TUBB3 and MAP2 that indicates more differentiated neurons was detected in NGLY1-deficient COs at day 80. *Red dots:* the technical replicates of mass spectrometry for each biological replicate of NGLY1-functional (C6 and P) COs. *Blue dots:* the technical replicates of mass spectrometry for each biological replicate of NGLY1-deficient (C3 and C4) COs. *Blue asterisks:* proteins with reduced abundance commonly found in day-40 and day-80 COs with NGLY1 deficiency. *Red asterisks:* proteins with increased abundance commonly found in day-40 and day-80 COs with NGLY1 deficiency. "*ND" highlighted in blue:* proteins non-detectable in NGLY1-deficient COs. "*ND" highlighted in red:* proteins non-detectable in NGLY1-functional COs. *Purple shading:* differential abundance with borderline significance (p=0.052).

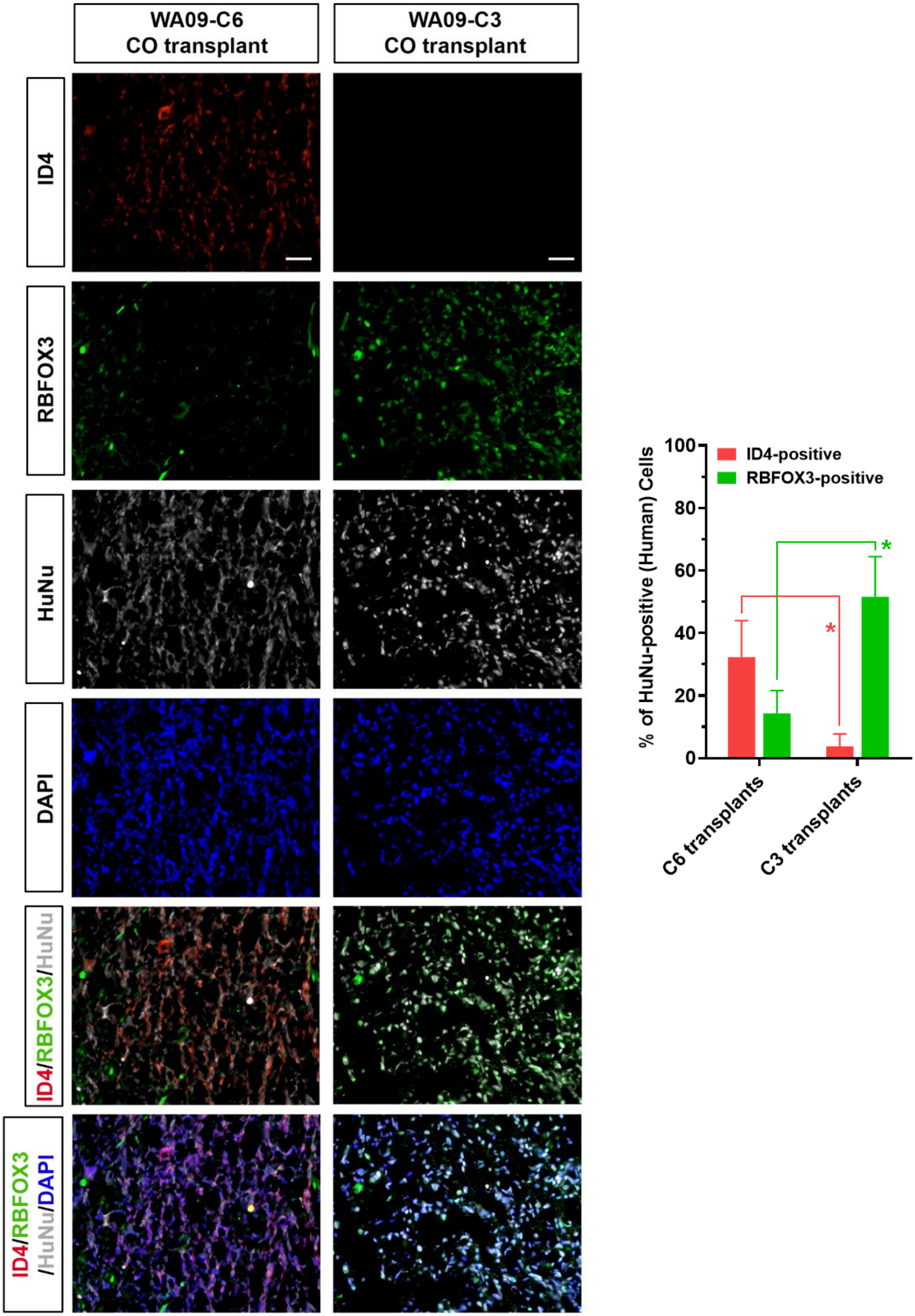
WA09-C6
CO transplant
WA09-C3
CO transplant
ID4
RBFOX3
HuNu
DAPI
ID4/RBFOX3/HuNu
ID4/RBFOX3
/HuNu/DAPI
% of HuNu-positive (Human) Cells
100
80
60
40
20
0
ID4-positive
RBFOX3-positive
*
*
C6 transplants
C3 transplants

Fig. S8. The presence of ID4-positive and RBFOX3-positive human cells detected by immunofluorescence staining in NGLY1-functional and -deficient CO transplants developed in the mouse brain. WA09-C6 (NGLY1-functional) and WA09-C3 (NGLY1-deficient) COs at day 15 of development were transplanted into the cerebral cortex of 8-week-old mice for further development and maturation. Tissue harvesting was performed after 50 post-implementation days. *Left panel:* Representative images from 5 randomly selected sections of different regions adjacent to the mouse brain tissue in each indicated CO transplant. Scale bar, 50 µm. *Right panel:* The percentages of ID4-positive and RBFOX3-positive cells in >800 counted human cells from each transplant. All data were presented as mean ± standard deviation (n=3 transplants for each type, *p<0.05, t-test).

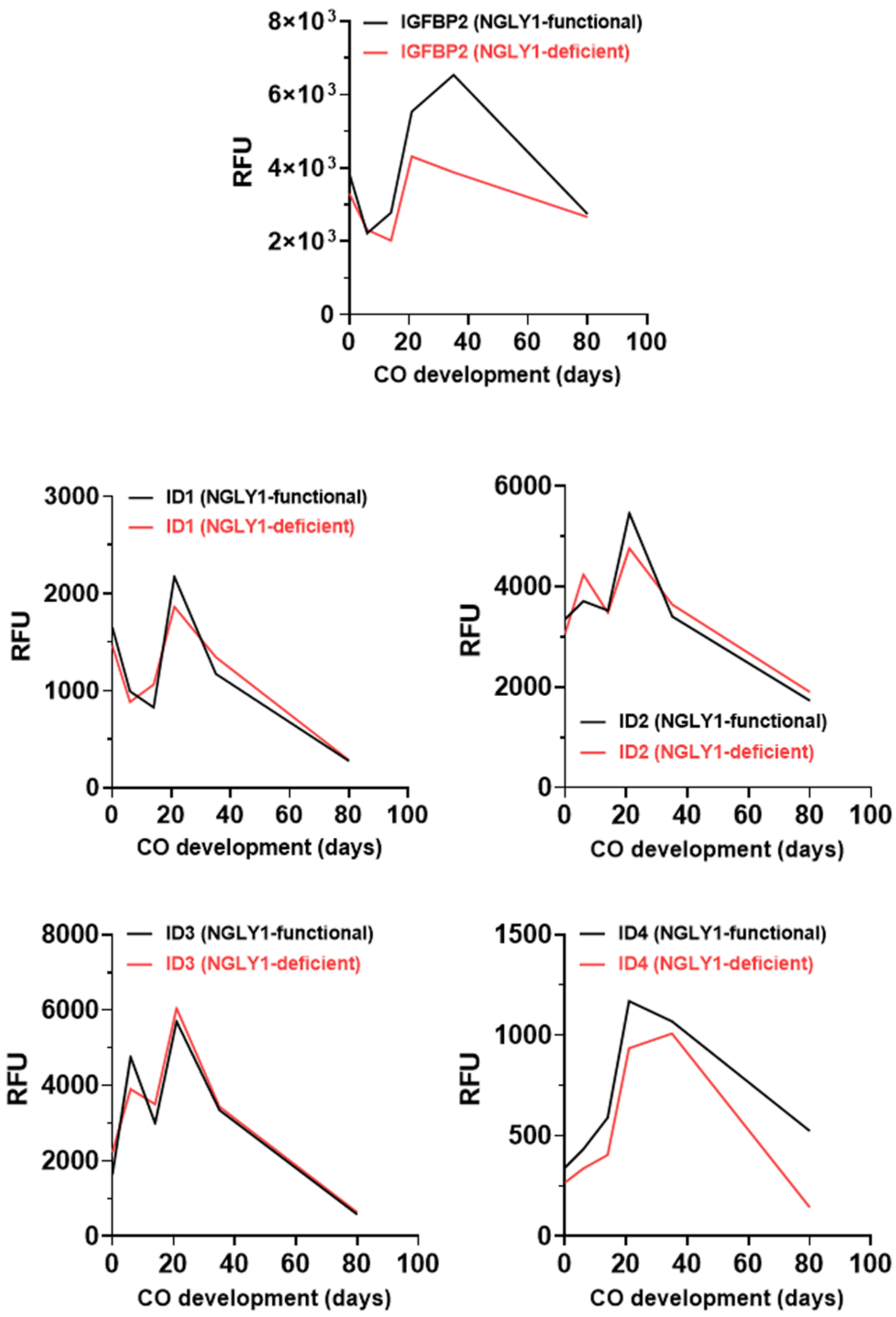
IGFBP2 (NGLY1-functional)
IGFBP2 (NGLY1-deficient)
RFU
8×10³
6×10³
4×10³
2×10³
0
0 20 40 60 80 100
CO development (days)
ID1 (NGLY1-functional)
ID1 (NGLY1-deficient)
3000
2000
1000
0
ID2 (NGLY1-functional)
ID2 (NGLY1-deficient)
6000
4000
2000
ID3 (NGLY1-functional)
ID3 (NGLY1-deficient)
8000
6000
4000
2000
ID4 (NGLY1-functional)
ID4 (NGLY1-deficient)
1500
1000
500

Fig. S9. The expression patterns of the *IGFBP2*, *ID1*, *ID2*, *ID3,* and *ID4* genes revealed by microarray analysis in NGLY1-functional and -deficient COs at different time points during 80 days of development. COs were developed from NGLY1-functional and -deficient WA09 hESCs. Samples were collected at either day 0, 6, 14, 21, 35, or 80 for microarray analysis. Average relative fluorescence units (RFUs, *n*=4 from each NGLY1 condition at each time point) associated with the indicated gene probes in the normalized array data were plotted along the course of CO development.

Vid. S1. The sweeping video of all optical-section images acquired from a whole NGLY1-functional (WA09-C6) CO by light-sheet-microscopy. *Green:* SATB2. *Red:* BCL11B. *White:* MAP2. The CO was collected after 100 days of development.

Vid. S2. The sweeping video of all optical-section images acquired from a whole NGLY1-deficient (WA09-C3) CO by light-sheet-microscopy. *Green:* SATB2. *Red:* BCL11B. *White:* MAP2. The CO was collected after 100 days of development.

Table S1. The list of cultured cells used in the studies		
**Sample Name**	**Registry Name[a]**	**Note[b]**
**Human embryonic stem cells (hESCs)**		
WA09	WA09	Obtained from the WiCell Stem Cell Bank; feeder cell-free culture on Matrigel, passaged using EDTA-based hPSC passaging solution (Thermo Fisher Scientific)
WA09-C6	WA09	The NGLY1 editing-escaped clone of WA09 hESCs that retain NGLY1 expression; feeder cell-free culture on Matrigel, passaged using EDTA-based hPSC passaging solution (Thermo Fisher Scientific)
WA09-C3	WA09	The NGLY1-edited clone of WA09 hESCs that lost NGLY1 expression; feeder cell-free culture on Matrigel, passaged using EDTA-based hPSC passaging solution (Thermo Fisher Scientific)
WA09-C4	WA09	The NGLY1-edited clone of WA09 hESCs that lost NGLY1 expression; feeder cell-free culture on Matrigel, passaged using EDTA-based hPSC passaging solution (Thermo Fisher Scientific)
**Human induced pluripotent stem cells (hiPSCs)**		
NGLY1Pt1i-507	N/A	Sendai virus-mediated reprogramming in NGLY1-deficient patient's dermal fibroblasts (GM25990); feeder cell-free culture on Matrigel, passaged using EDTA-based hPSC passaging solution
NGLY1Pt1i-508	N/A	Sendai virus-mediated reprogramming in NGLY1-deficient patient's dermal fibroblasts (GM25990); feeder cell-free culture on Matrigel, passaged using EDTA-based hPSC passaging solution
NGLY1Pt1i-509	N/A	Sendai virus-mediated reprogramming in NGLY1-deficient patient's dermal fibroblasts (GM25990); feeder cell-free culture on Matrigel, passaged using EDTA-based hPSC passaging solution
NGLY1Pt2i-502	N/A	Sendai virus-mediated reprogramming in NGLY1-deficient patient's dermal fibroblasts (GM26607); feeder cell-free culture on Matrigel, passaged using EDTA-based hPSC passaging solution
**Cancer cells**		
MALME3M_shRNA645	N/A	Human melanoma cells with the inducible expression of shRNA targeting NGLY1, cultured using DMEM medium containing 10% FBS, enzymatic passaged using trypsin-EDTA
**Cells used for reprogramming**		
HDF (GM25990)	N/A	Human dermal fibroblasts derived from the skin biopsy sample of a female patient with NGLY1 deficiency, cultured using DMEM medium containing 10% FBS, obtained from Coriell Biorepository [The patient is compound heterozygous for two mutations in the NGLY1 gene. A mutation of in-frame trinucleotide deletion that begins at position 3:25775416 (exon 8 of *NGLY1*) and leads to 1205_1207del in cDNA and a stop-gain mutation at position 3:25761670 (exon 11 of *NGLY1*, 1570C>T in cDNA, R542X in protein) are identified.]
HDF (GM26607)	N/A	Human dermal fibroblasts derived from the skin biopsy sample of a male patient with NGLY1 deficiency, cultured using DMEM medium containing 10% FBS, obtained from Coriell Biorepository [A homozygous mutation of 1201A>T in the NGLY1 cDNA (exon 8) that leads to R401X in protein is identified in this patient.]

a. Name of cell line listed in the University of Massachusetts (UMass) International Stem Cell Registry

b. Somatic cell type, reprogramming method, culture condition, source of cells

N/A: not applicable

Table S2. The list of primary antibodies and lectin used in the studies		
**Antibody/Lectin Name**	**Catalog Number**	**Sources**
**Antibodies used in IHC or fluorescence staining**		
TRA-1-81	09-0011	Stemgent
POU5F1	2840	Cell Signaling Technology
NANOG	MABD24	Millipore Sigma
TUBB3	MRB-435P	Biolegend (formerly Covance)
Smooth Muscle Actin (SMA)	MAB1420	R&D Systems
SOX17	AF1924	R&D Systems
SATB2	ab34735	Abcam
BCL11B (CTIP2)	ab18465	Abcam
MAP2	ab34735	Abcam
FOXG1	ab18259	Abcam
ID4	BCH-9/82-12	Biocheck
RBFOX3	ABN90P	Millipore Sigma
Human Nuclear Antigen	MAB1281B	Millipore Sigma
**Antibodies used in immunoblotting**		
NGLY1	HPA036825	Millipore Sigma
Phospho-STAT3 (Tyr705)	9145	Cell Signaling Technology
STAT3	4904	Cell Signaling Technology
ATF4	11815	Cell Signaling Technology
HES1	11988	Cell Signaling Technology
BCL11B	12120	Cell Signaling Technology
SATB2	ab34735	Abcam
ACTIN	08691001	MP Biomedicals
NEUROD2	ab109406	Abcam
ATF4	11815	Cell Signaling Technology
GADD153	NB600-1335	Novus Biological
TTR	ab75815	Abcam
IGFBP2	AF674	R&D Systems
ID4	EPR22323-36	Abcam
LRP2	ab76969	Abcam
Phospho-ERK 1/2 (Thr202/Tyr204)	4370	Cell Signaling Technology
ERK 1/2	4696	Cell Signaling Technology
**Lectin used in fluorescence staining**		
UEA-I	FL-1061	Vector Laboratories

Provision of original western blots in the article (Figure 1)

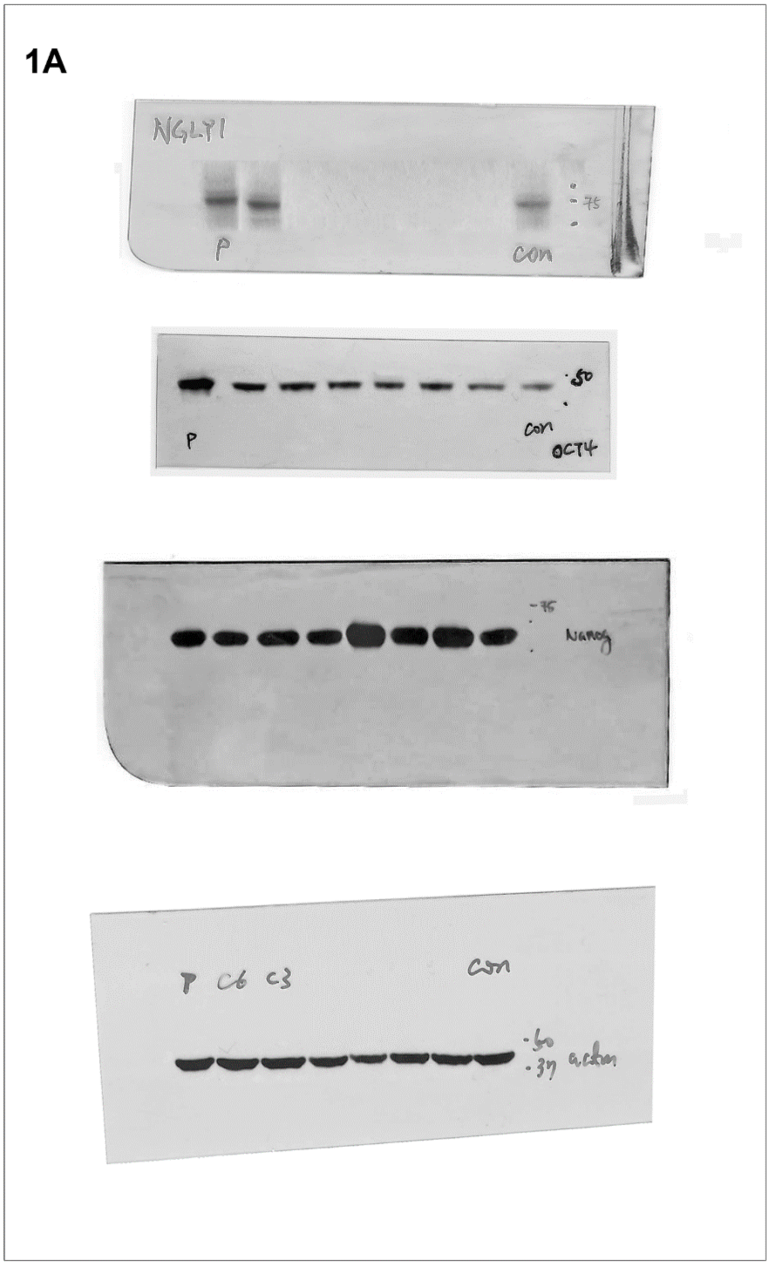

Provision of original western blots in the article (Figure 2)

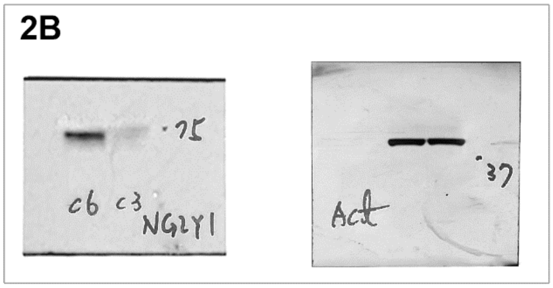

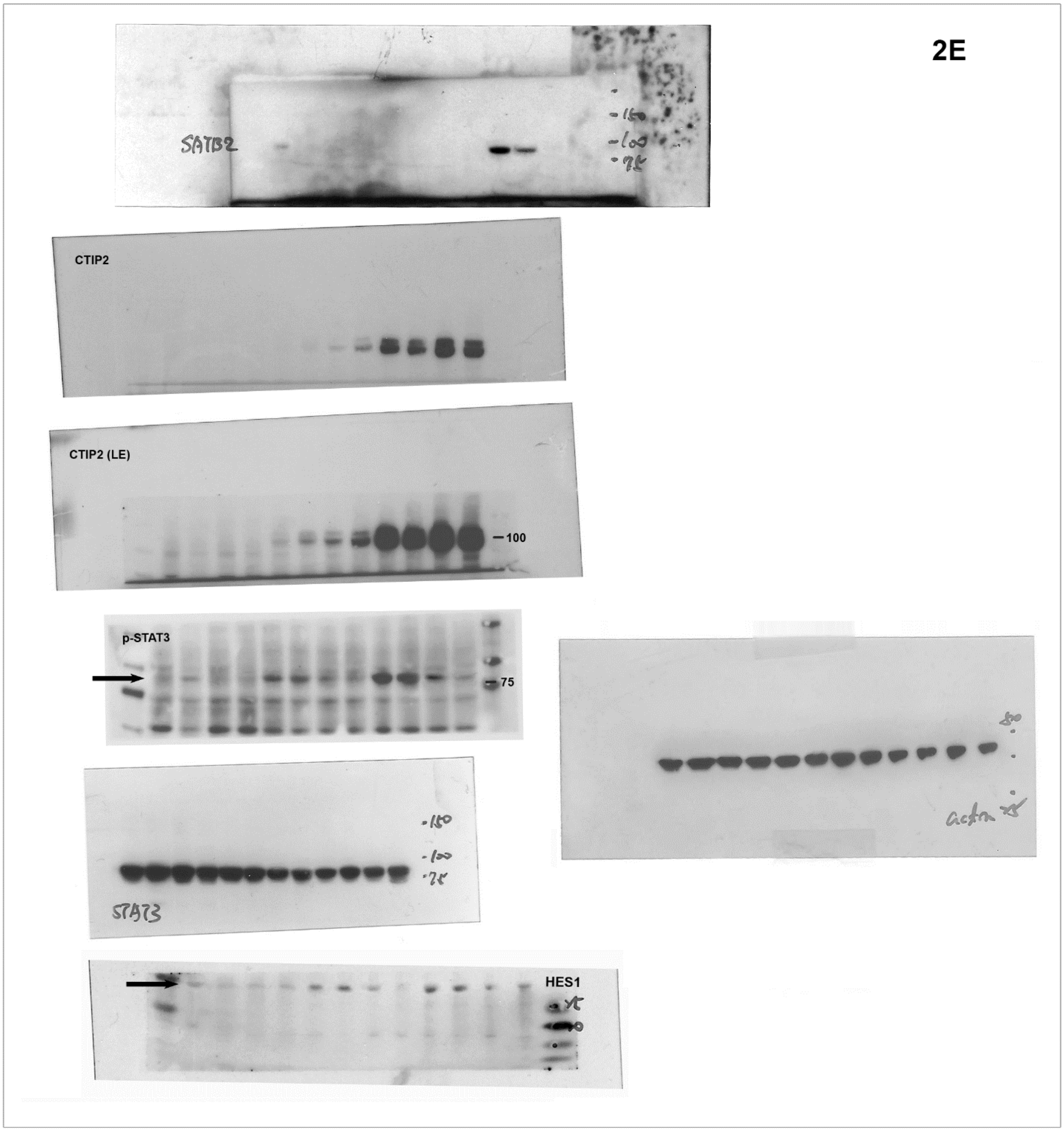

Provision of original western blots in the article (Figure 3)

3A

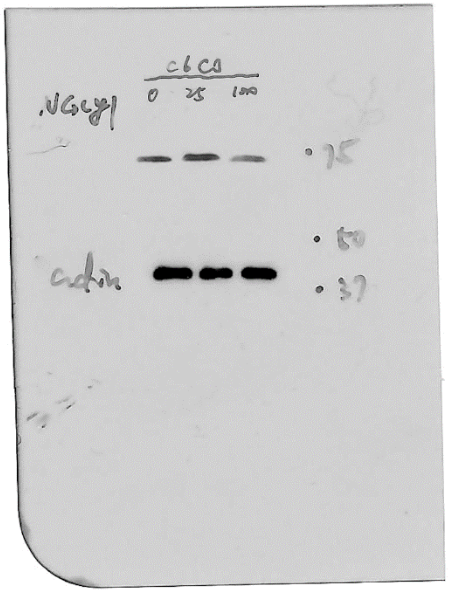

3C

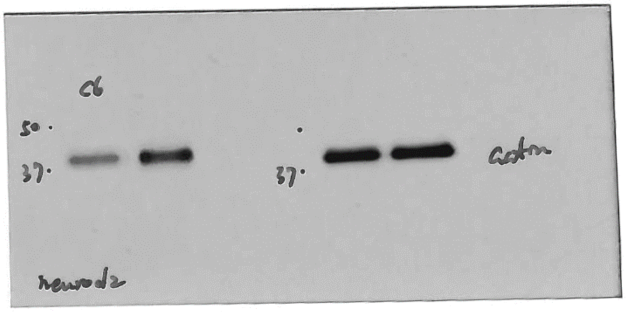

3D

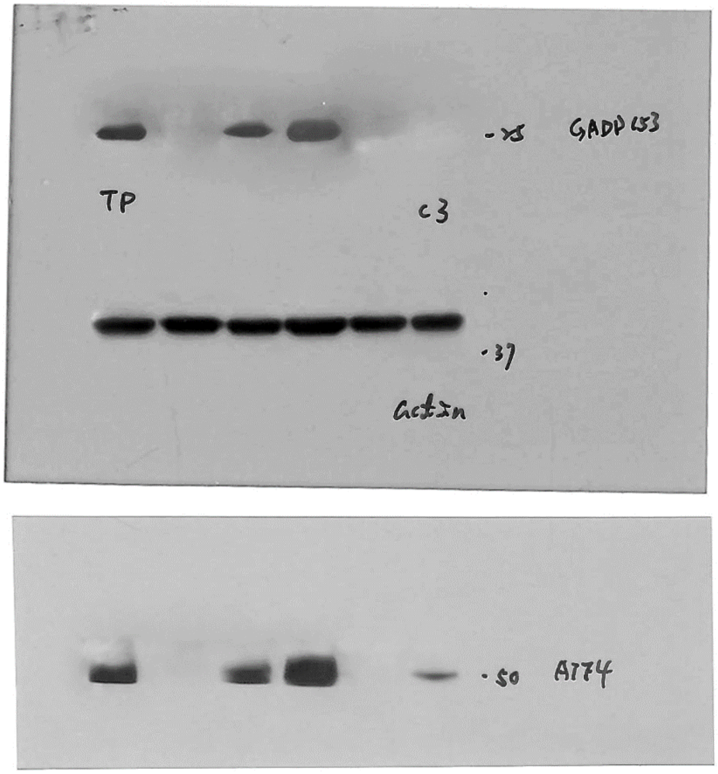

Provision of original western blots in the article (Figure 4)

4G

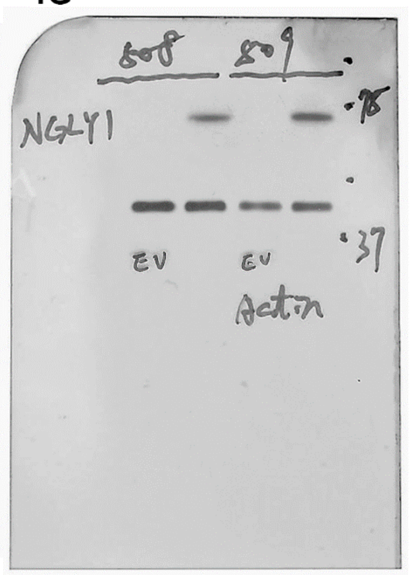

Provision of original western blots in the article (Figure 5)

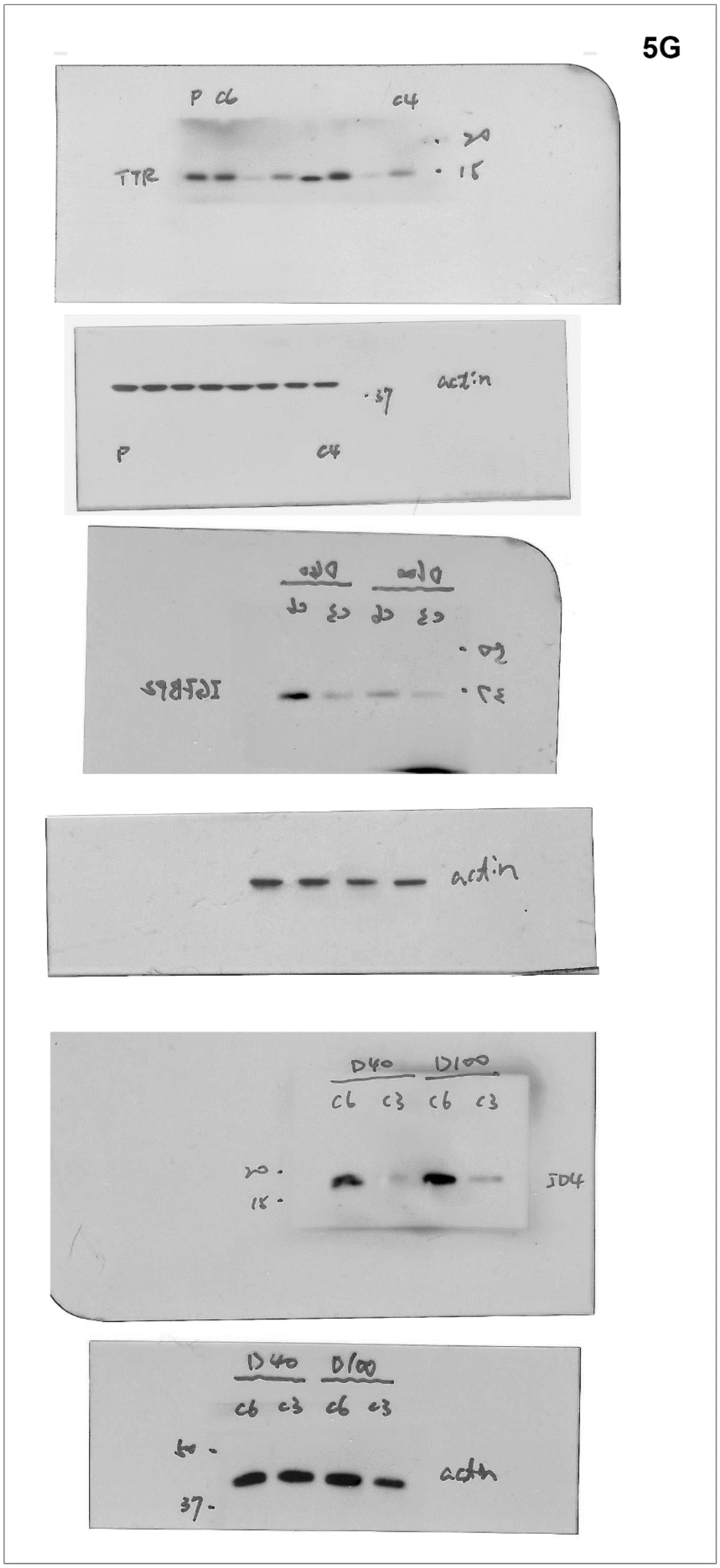

Provision of original western blots in the article (Figure 6)

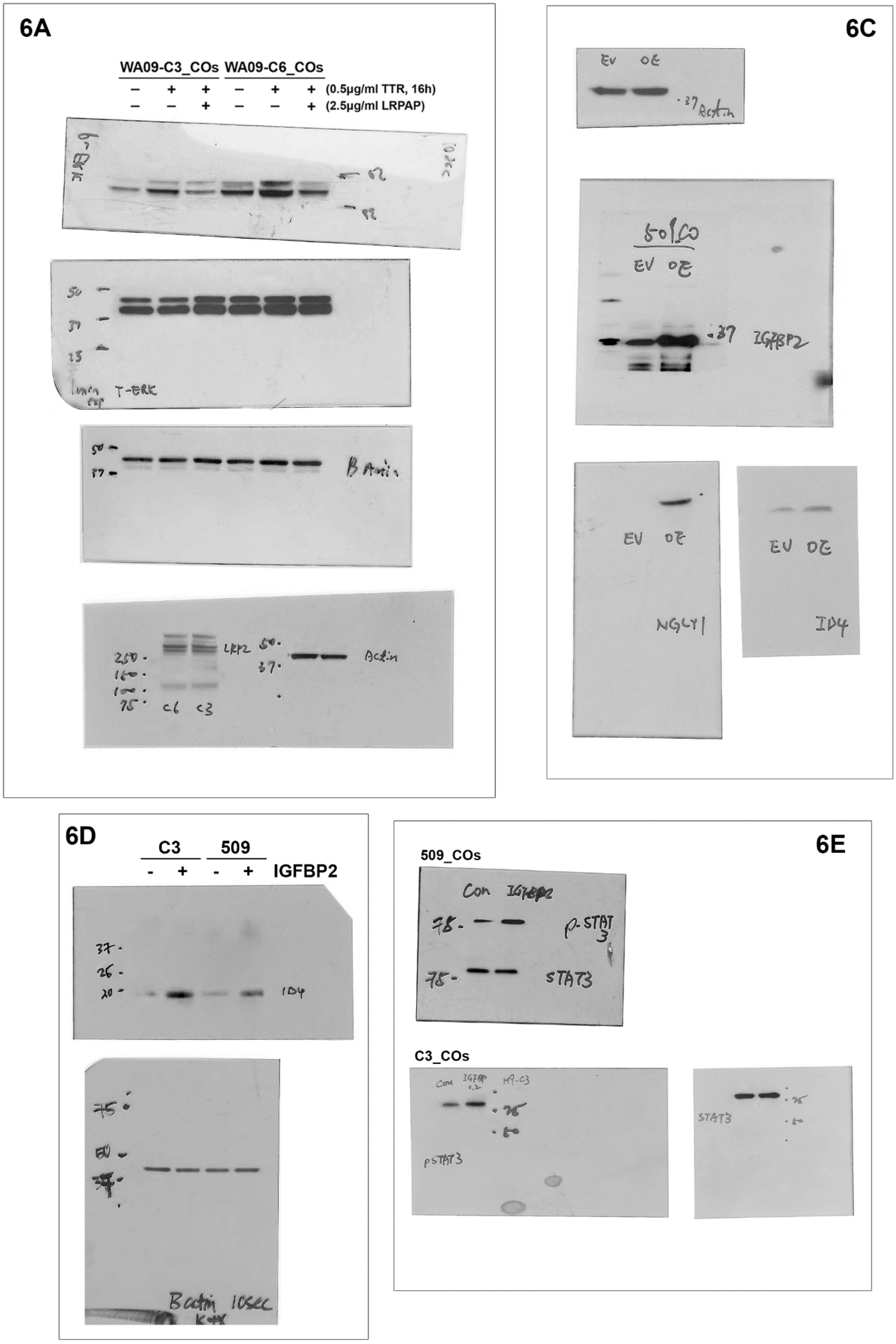

Supplement: Supplementary file 1 — Supplemental Methods and Data [file 41419_2022_4693_MOESM1_ESM.pdf]
